# Linker Chemistry and Connectivity Fine-Tune the Immune Response and Kinetic Solubility of Conjugated NOD2/TLR7 Agonists

**DOI:** 10.1021/acs.bioconjchem.4c00321

**Published:** 2024-10-10

**Authors:** Špela Janež, Samo Guzelj, Žiga Jakopin

**Affiliations:** Faculty of Pharmacy, University of Ljubljana, SI-1000 Ljubljana, Slovenia

## Abstract

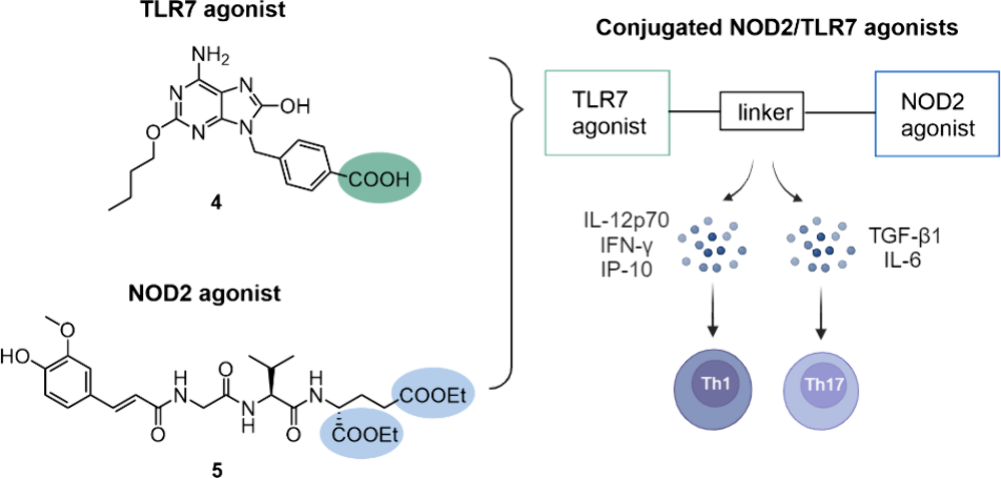

There is a growing interest in developing novel immune potentiators capable of eliciting a cellular immune response. We tackle this challenge by harnessing the synergistic cross-activation between two innate immune receptors—the nucleotide-binding oligomerization domain-containing protein 2 (NOD2) and Toll-like receptor 7 (TLR7). Herein, we investigate the structure–activity relationship of a series of novel conjugated NOD2/TLR7 agonists incorporating a variety of flexible aliphatic, poly(ethylene glycol)-based and triazole-featuring linkers. Our findings reveal potent immune-enhancing properties of conjugates in human primary peripheral blood mononuclear cells, characterized by a Th1/Th17 polarized cytokine response. Importantly, we demonstrate that both the chemistry of the linker and the site of linkage affect the immune fingerprint and the kinetic solubility of these conjugated agonists. These results shed further light on the immunostimulatory potential of NOD2/TLR7 cross-activation and provide insights for designing innovative immune potentiators.

Vaccines have significantly reduced the incidence of infections and even eradicated once devastating diseases including diphtheria and smallpox.^1^ Despite the overwhelming success of vaccination there are still many diseases that lack effective vaccines due to insufficient immune responses and/or weak immunological memory.^2,3^ To overcome these challenges, innate immune agonists targeting antigen-presenting cells, such as dendritic cells (DCs), are often introduced into vaccines as adjuvants.^1^ Their adjuvant effect is achieved indirectly by activation of innate immunity - by engaging pattern recognition receptors (PRRs), such as nucleotide-binding oligomerization domain (NOD)-like receptors (NLRs) and Toll-like receptors (TLR), with their agonists.^3,4^

Albeit single PRR agonists, such as TLR agonists, are at the forefront of adjuvant development,^1^ they are not always sufficient to elicit effective systemic immune responses. However, the cross-talk between different innate immune receptors not only permits signal amplification but also determines the type of the response induced (e.g., cellular or humoral).^5^ A prime example is one of the most effective vaccines, the yellow fever vaccine, which engages the immune system via synergistic stimulation of multiple PRRs.^1^ The concept of using multiple agonists is therefore key to achieving a robust systemic response, and specific combinations have already been employed in vaccine adjuvants used in the clinic (e.g., adjuvant systems AS01, AS04).^6^ The simultaneous activation of distinct PRRs by a mixture of agonists can elicit a more efficient and broader immune system response, while the latter can be further enhanced by covalent chemical linkage of such combinations of PRR agonists.^1^ In line with this notion, we and others have demonstrated that covalent conjugation of PRR agonists (e.g., TLR2/TLR7, TLR4/TLR9, TLR2/TLR9, NOD2/TLR2, NOD2/TLR7) can achieve a superior immune response compared to mixtures of unconjugated agonists, reduce the adjuvant dosing and improve the safety profile due to restricted systemic diffusion.^7−13^

TLR7 and TLR8 are intracellular endosomal receptors, which detect single-stranded RNA.^6,14,15^ In humans, TLR7 is mainly expressed in plasmacytoid DCs and contributes to their maturation and differentiation, which in turn, increases the ability for antigen presentation.^16^ Conversely, TLR8 has a complementary expression pattern, being mainly expressed in monocytes and myeloid DCs, directing their activation toward a strong cellular Th1 immune response.^17^ NOD2 is an intracellular innate immune receptor, widely expressed in immune cells, which recognizes fragments of bacterial peptidoglycan resembling muramyl dipeptide (MDP). Importantly, besides activating nuclear factor κB (NF-κB) and mitogen activated protein kinase signaling pathways,^18,19^ NOD2 agonists also trigger the maturation and activation of DCs.^20^ Co-engagement of NOD2 and TLR7 receptors synergistically augmented cytokine release from peripheral blood mononuclear cells (PBMCs) and macrophages,^21,22^ while a combination of the NOD2 agonist MDP and TLR7/8 agonist resiquimod showed superadditive DC activation and generation of humoral and cellular immune responses in vivo.^9,23,24^

Our previous efforts led to the discovery of two conjugated NOD2/TLR7 agonists (**1** and **2**, Figure 1), constructed by linking our flagship desmuramylpeptide NOD2 agonist **5** and a purine scaffold-based TLR7 agonist **4**. They showed potent immunostimulatory activities in vitro as well as the ability to elicit a robust antigen-specific immune response *in vivo* with a Th1-polarized profile.^10^ Similar activities have been reported for the NOD2/TLR7 agonist featuring MDP (**3**, Figure 1), which also induced mucosal immune response.^9^ Large molecules, including TLR agonist-featuring conjugates, sometimes suffer from poor aqueous solubility, which hinders their systemic delivery. Classical strategies employed in small molecule design cannot be directly applied to conjugates. Namely, the biological activity and physicochemical properties of the conjugates are strongly influenced by the linker connecting the ligands.^25^ The most commonly used motifs for linker structures are linear alkyl- and polyethylene glycol (PEG)-based spacers of various lengths. Typically, shorter linkers composed of 2–3 PEG units produce the most optimal compounds in terms of balancing aqueous solubility and cell permeability.^26^ However, the best linker types for a given conjugate are also likely to be scaffold dependent.^26^ Subtle changes in the connectivity of the linker groups to the ligands (i.e., linkage sites) can also impact activity and solubility.^25,27,28^

**Figure 1:**
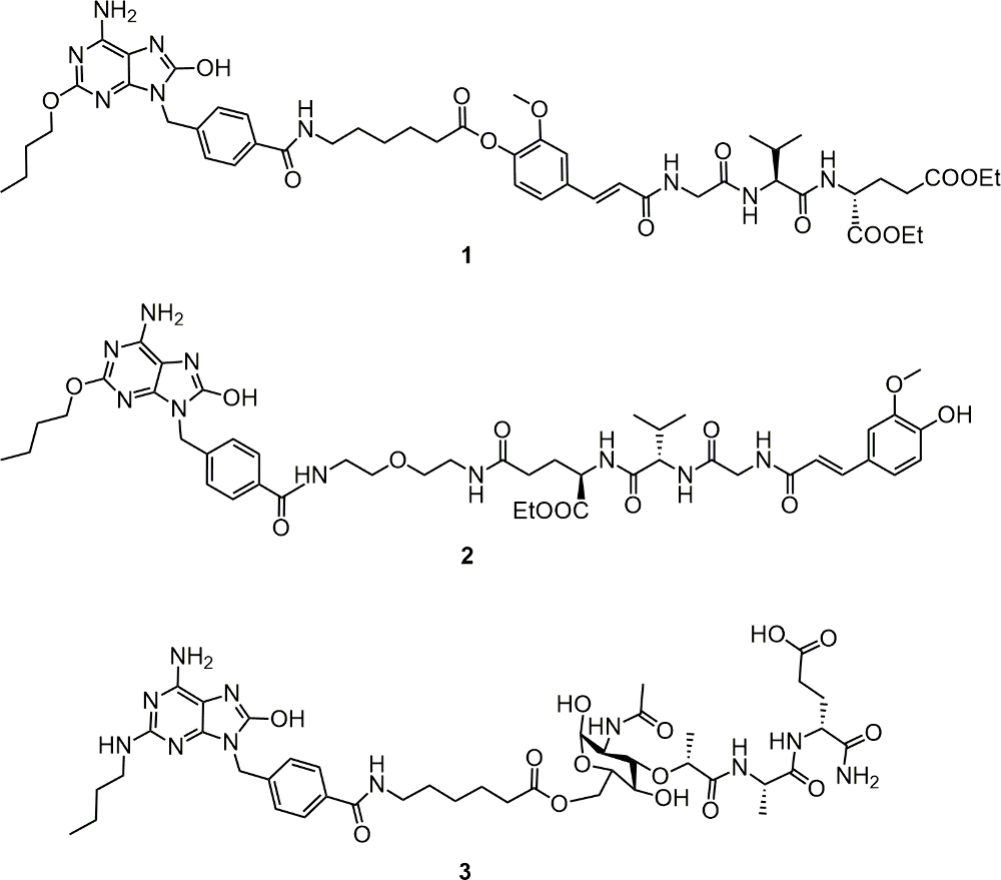
Chemical structures of conjugated NOD2/TLR7 agonists **1**, **2**,^10^ and **3**.^9^

In this study, we continue our systematic exploration to ascertain whether and how the use of diverse linkers as well as distinct linkage sites on the NOD2 agonist (Figure 2) can impact the NOD2/TLR7 conjugates’ immune potentiating activity and solubility. Further, given that mixed TLR7/8 agonists are capable of highly efficient and broad immune system activation, we also assembled conjugated NOD2/TLR7/8 employing various TLR7/8 agonists–resiquimod and its amino derivative (Figure 2).

**Figure 2:**
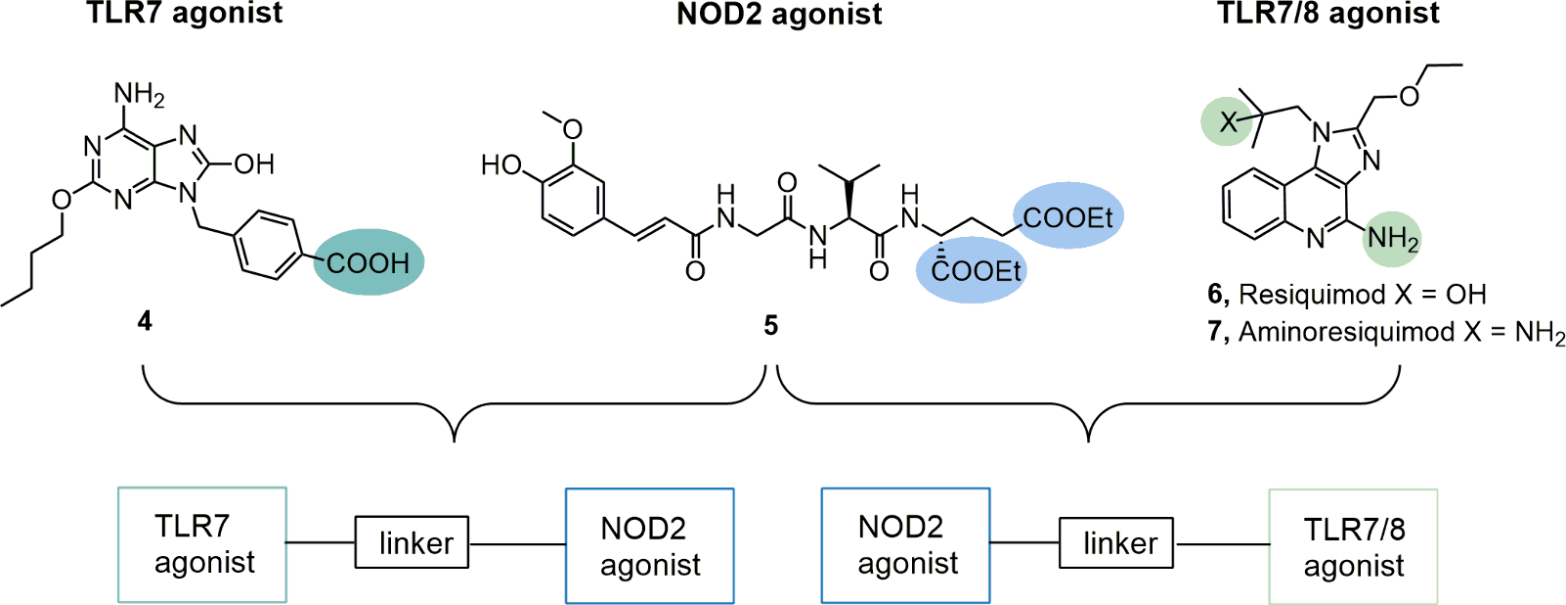
Design of novel conjugated NOD2/TLR7 and NOD2/TLR7/8 agonists.

We first set out to prepare a series of conjugated NOD2/TLR7 agonists using a variety of flexible aliphatic, PEG-based and triazole-featuring linkers attached to the γ-carboxyl group of the NOD2 agonist while retaining the amide to amide, or alternatively introducing ester to amide, bonding chemistry. The synthetic strategy (shown in Scheme 1) involved the attachment of the TLR7 agonist **4** to Boc-protected linkers **8**, **9**, **10** and acetyl-protected linker **13** (prepared as described in SI) using the 1-[(1-(cyano-2-ethoxy-2-oxoethylideneaminooxy)-dimethylamino-morpholinomethylene)]-methanaminium hexafluorophosphate (COMU)-mediated coupling to afford **18**, **19**, **20** and **21**. Acidolytic cleavage of the Boc-protecting groups of **18**, **19**, **20** and subsequent coupling to the free γ-carboxyl group of the tetrahydropyranyl-protected NOD2 agonist **5a** produced the final conjugates **22**, **23**, **24**, respectively (the tetrahydropyranyl group underwent acidolytic removal during workup). Unfortunately, upon cleavage of the acetyl protecting group of **21**, the subsequent coupling to NOD2 agonist **5a** did not afford the desired conjugate **25**. In order to investigate whether the linker attachment site on the NOD2 agonist also impacts the pharmacodynamic properties, a positional isomer of **22**, namely conjugate **26** featuring an α-carboxyl group linkage between the NOD2 agonist and the TLR7 agonist moiety was constructed by coupling of NOD2 agonist **5b** carrying a free α-carboxyl group (prepared as described in SI) with the linker-decorated TLR7 agonist **18**.

**Scheme 1:**
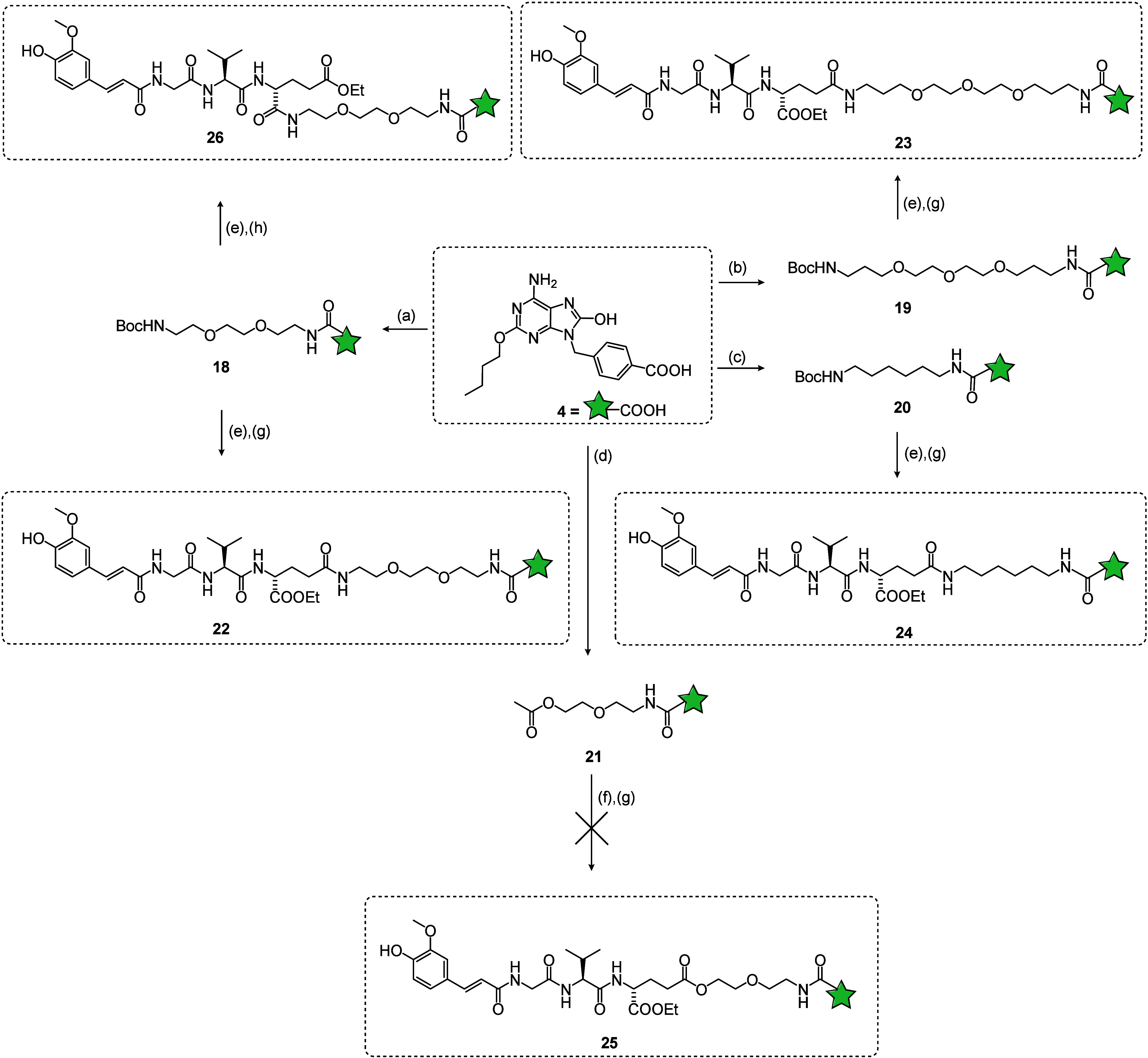
Synthesis of Conjugated NOD2/TLR7 Agonists **22**-**26** Reagents and conditions: (a) **8**, COMU, DIPEA, DMF, rt.; (b) **9**, COMU, DIPEA; DMF, rt.; (c) **10**, COMU, DIPEA, DMF, rt.; (d) **13**, COMU, DIPEA, DMF, rt.; (e) TFA/DCM (1:5), rt.; (f);K_2_CO_3_, MeOH (g) **5a**, COMU, DIPEA, DMF, rt; (h) **5b**, COMU, DIPEA, DMF, rt.

Next, we wanted to explore alternative viable avenues for the final step of conjugate assembly. To that end, we relied on the click-chemistry methodology, which has previously been successfully utilized in numerous (bio)conjugates.^29,30^ As depicted in Scheme 2, the azide derivative of TLR7 agonist **27** was synthesized by 1-[bis(dimethylamino)methylene]-1H-1,2,3-triazolo[4,5-*b*]pyridinium 3-oxide hexafluorophosphate (HATU)-mediated coupling of 4 with azido-PEG_3_ amine, while NOD2 agonist **5a** was reacted with propargylamine to generate the alkyne **28**. The triazole-incorporating conjugate **29** was then assembled by merging the alkyne handle-decorated NOD2 agonist **28** and an azide functionality installed on the TLR7 agonist-linker adduct **27** under mild conditions using copper(II) sulfate pentahydrate and sodium ascorbate.

**Scheme 2:**
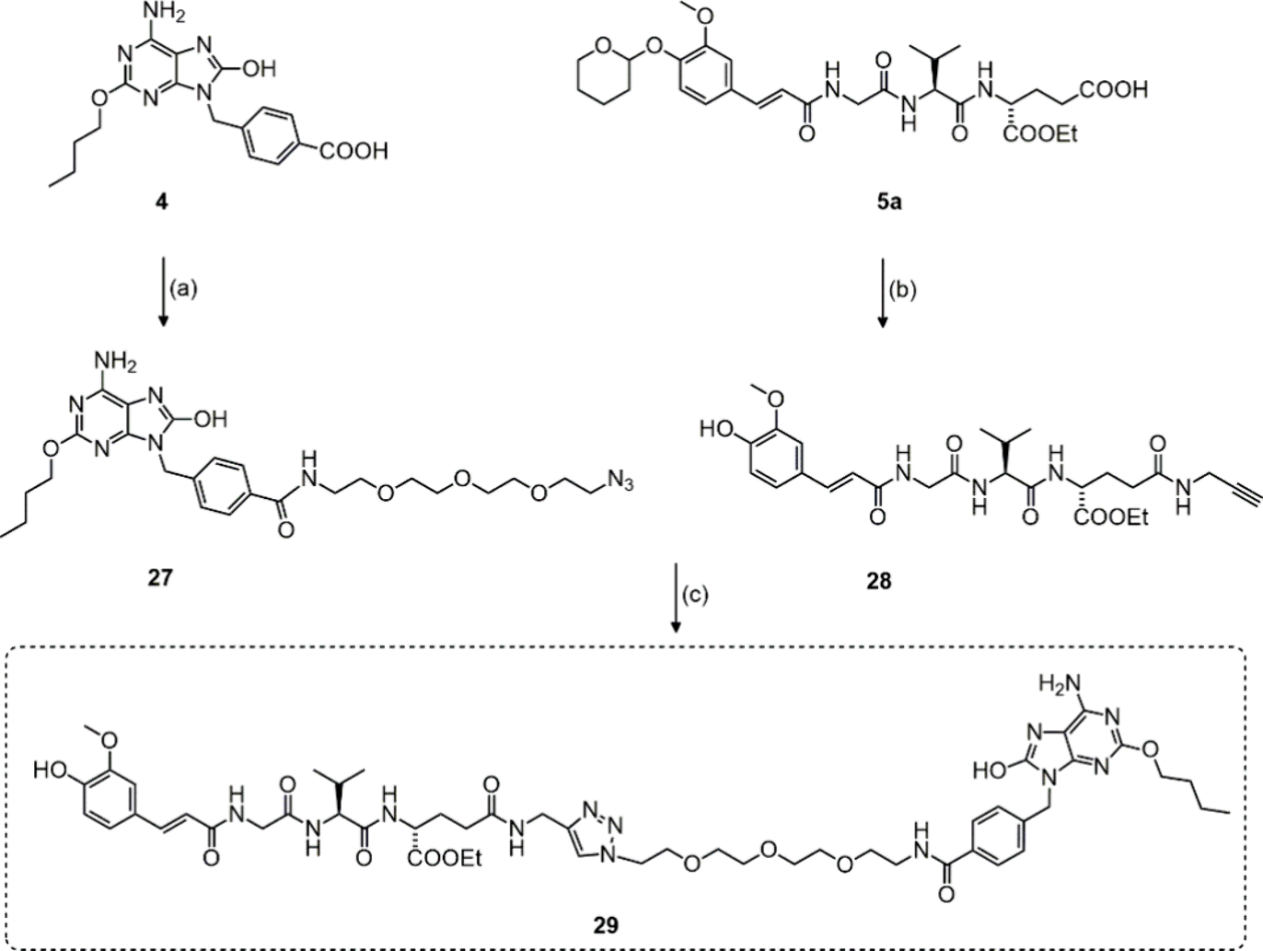
Assembly of Conjugated NOD2/TLR7 Agonist **29** Reagents and conditions: (a) propargyl amine, COMU, DIPEA, DMSO, rt., 2h, (b) azido-PEG_3_-amine, HATU, DIPEA, DMF, rt., (c) CuSO_4_·5H_2_O, Na-ascorbate, DMF, rt.

Lastly, in order to extend the structure–activity relationship (SAR) analysis by introducing TLR8 agonism into the equation, the NOD2 agonist was covalently linked with the commercially available TLR7/8 agonist resiquimod and its closely related amino derivative (shown in Scheme 3). The γ-carboxyl group of the d-glutamyl residue in NOD2 agonist has previously been shown as optimal for linkage to TLR7 agonists, therefore we exploited this attachment site also for the construction of chimeric NOD2/TLR7/8 agonists. Resiquimod (**6**) was coupled with the 6-(Boc-amino)hexanoic acid (**11**) linker to generate the intermediate **30**. The ensuing acidolytic cleavage of the Boc-protecting group produced the free amine, which was immediately coupled to the NOD2 agonist **5a** thus giving the conjugated NOD2/TLR7/8 agonist **32**. In parallel, the amino derivative of resiquimod **7**(31) was coupled with N-benzyloxycarbonyl-6-aminohexanoic acid (**12**) to yield the intermediate **31**. Debenzylation of **31** with hydrogenation over palladium/carbon produced the free amine, which was then coupled with the NOD2 agonist **5a** to afford the alternative conjugated NOD2/TLR7/8 agonist **33**.

**Scheme 3:**
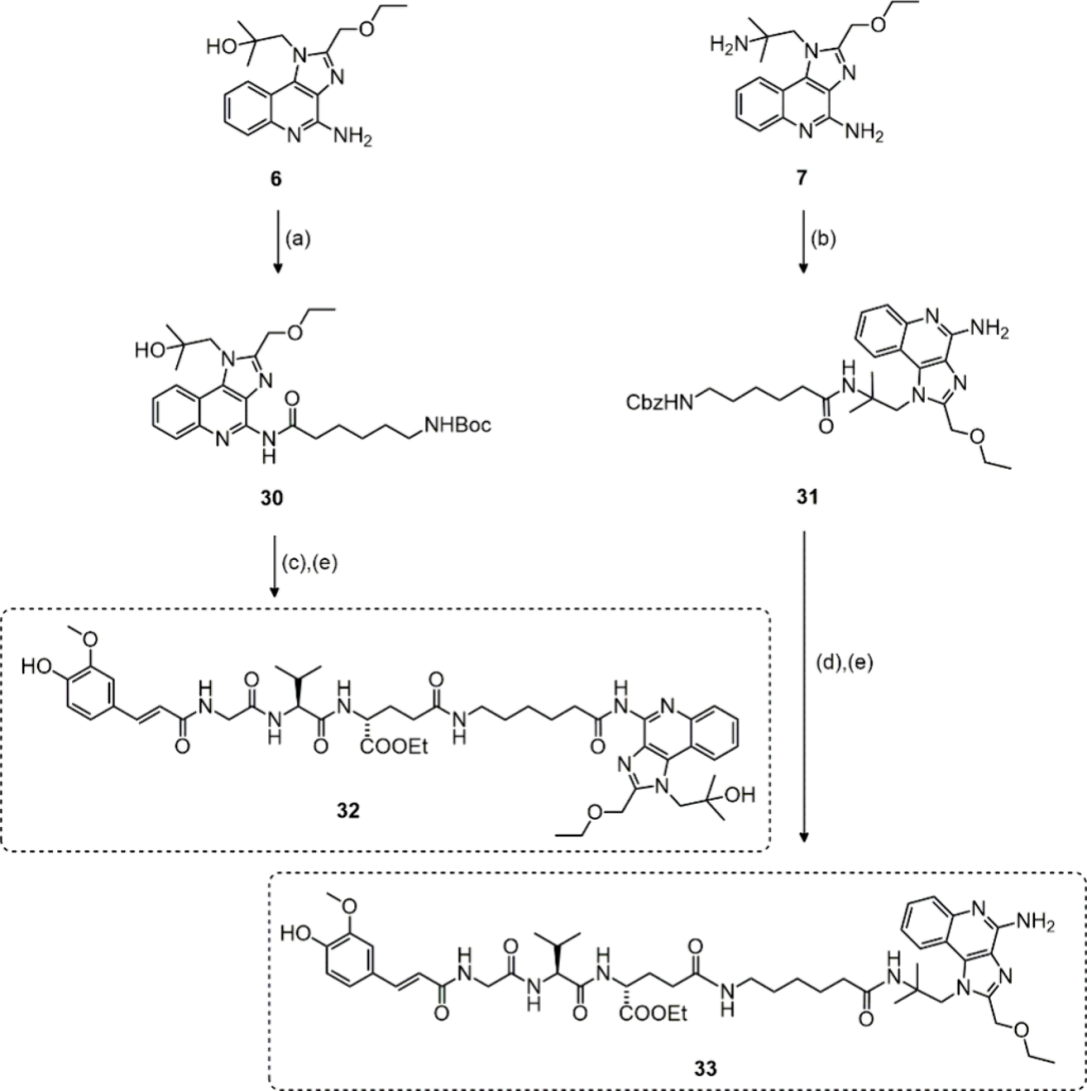
Synthesis of Conjugated NOD2/TLR7/8 Agonists **32** and **33** Reagents and conditions: (a) **11**, HATU, DIPEA, DMF, rt.; (b) **12**, HATU, DIPEA, DMF, rt.; (c) TFA/DCM (1:5), rt.; (d) H_2_, Pd/C, EtOH; (e) **5a**, HATU, DIPEA, DMF, rt.

The building blocks and synthesized conjugates were initially assessed for their receptor-specific activities toward NOD2, TLR7 and TLR8 using the commercially available HEK-Blue NOD2, TLR7 and TLR8 reporter cell lines. These cell lines were derived from HEK293 cells by cotransfection of human NOD2/TLR7/TLR8 and an NF-κB-inducible secreted embryonic alkaline phosphatase (SEAP). Activation of NOD2/TLR7/TLR8, and subsequently of NF-κB, leads to the expression and secretion of SEAP, the levels of which can be quantified colorimetrically. The results are shown in Table 1. Following confirmation of noncytotoxicity through the established 3-(4,5-dimethylthiazol-2-yl)-5-(3-carboxymethoxyphenyl)-2-(4-sulfophenyl)-2H-tetrazolium (MTS) assay (Figure S1), activity assays revealed that the building blocks **4**, **5**, resiquimod (**6**), and **7** alone selectively activated solely their cognate receptors. Expectedly, most conjugates failed to activate NOD2 at the highest concentration tested with the exception of conjugates **24** (EC_50_ = 6.2 μM) and **32** (EC_50_ = 7.2 μM) that showed weak agonistic activity. HEK293 cells namely lack the enzymatic mechanism required for cleavage of the amide bond connecting the spacer and the NOD2 agonistic moiety, thus leaving γ-carboxylic acid of the d-glutamic acid moiety, which is essential for NOD2 binding, inaccessible.^32^ All five conjugated NOD2/TLR7 agonists **22**, **23**, **24**, **26** and **29** activated TLR7 in the midnanomolar range. The most potent compound **23** (EC_50_ = 165 nM) showed a 2.5-fold enhancement of TLR7 activity over **22** (EC_50_ = 419 nM) and **29** (EC_50_ = 391 nM), while **24** (EC_50_ = 238 nM) and **26** (EC_50_ = 247 nM) exhibited comparable potencies. Of the conjugated NOD2/TLR7/8 agonists, **33** elicited only minimal TLR7 activation (EC_50_ = 5.3 μM) at the highest concentration tested, while **32** was devoid of TLR7 agonistic activity. Similarly, neither **32** nor **33** were able to induce any TLR8 agonistic activity. Clearly, imidazoquinolines do not tolerate binding either to the side-chain amine nor to the amine positioned on the central aromatic ring without causing a drop in both TLR7 and TLR8 activities, which is in agreement with the results of amine-modified resiquimod derivatives in reporter assays.^33^ Again, this can be ascribed to HEK293 reporter cells not being capable of cleaving the amide bond between the spacer and the TLR7/8 agonistic moiety.

**Table 1:**
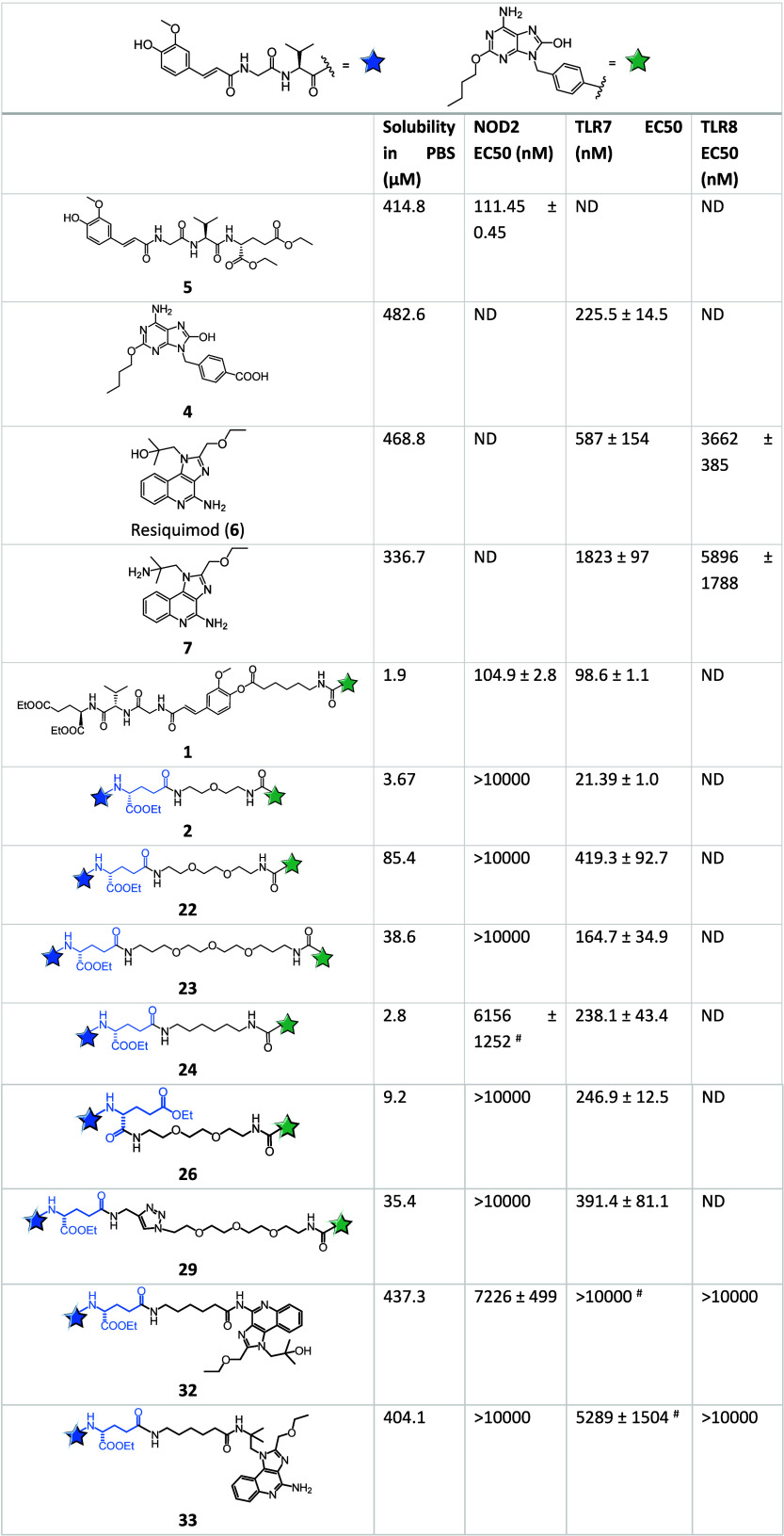
Kinetic solubility and NOD2, TLR7, and TLR8 agonistic activities of synthesized conjugates^a^

a EC_50_ values determined in HEK-Blue NOD2, TLR7, and TLR8 cells in at least three independent experiments with eight concentrations (1 nM to 10 μM). Data are mean ± SEM of three or two^#^ independent experiments. ND, not determined.

Second, the kinetic solubility of all individual building blocks (compounds **4**-**7**) and conjugates was determined (results are listed in Table 1) in order to highlight how fine-tuning of linker traits can impact this physicochemical property. Kinetic solubility refers to the maximum solubility of a compound when it precipitates from a supersaturated solution, which is metastable and not in equilibrium; it was measured using the method described by Hoelke et al.^34^ The length and composition of the linker as well as linkage site are important for activity and physicochemical characteristics of conjugates, which in turn affect their pharmacokinetic profiles. In general, the benzylic COOH was exploited exclusively as the linker attachment site in TLR7 agonist-featuring conjugates, while two potential linkage sites were considered in the structure of NOD2 agonist. The individual building blocks exhibited very good solubilities ranging from 337 to 483 μM. In conjugates **22**-**24** and **29**, the moieties were connected via the γ-COOH group of the NOD2 agonist. Prolongation of the flexible di(aminoethanol) linker in our flagship conjugate **2** (3.67 μM) by a single ethylene glycol unit resulted in almost 25-fold boost in solubility (conjugate **22**, 85.4 μM). On the other hand, conjugate **23** incorporating a linker composed of two aminopropanol and ethylene glycol units, exhibited a solubility of 38.6 μM. It has to be kept in mind that among the polyalkylethers, the structural requirements for aqueous solubility are poised toward the (−OCH_2_CH_2_−)_n_ unit while (−OCH_2_CH_2_CH_2_−)_n_ is not beneficial in this respect.^35^ The insertion of a 1,2,3-triazole moiety into the PEG-based linker had negligible effect on the kinetic solubility as exemplified by conjugate **29** (35.4 μM), which could partly be ascribed to a suboptimal lateral position within the linker.^36^ In line with our expectations, conjugate **24** that features a flexible aliphatic linker displayed a poor solubility of 2.8 μM, comparable to that of conjugate **1** (1.9 μM) featuring a linker of a similar nature (albeit connected to the phenol OH group). Finally, the linkage of NOD2 agonist to TLR7 agonist via the α-COOH group featured in conjugate **26** (9.2 μM) appears to have a deleterious effect on the solubility in direct comparison to its positional isomer of **22** (85.4 μM) that incorporates the same linker. Interestingly, in spite of the presence of a flexible aliphatic linker both conjugated NOD2/TLR7/8 agonists, **32** and **33**, exhibited very good kinetic solubilities of 437 and 404 μM, respectively.

Third, the immunostimulatory potential of synthesized conjugates was assessed in human primary peripheral blood mononuclear cells (PBMCs) (Figure 3, Supporting Table), which represent a diverse array of immune cell subpopulations, providing a more physiologically relevant system for studying concurrent NOD2/TLR7 or NOD2/TLR7/TLR8 activation. Overnight stimulation of PBMCs with individual NOD2 and TLR7 agonists or their mixture led to minor increases in production of proinflammatory cytokines, including IL-8, MCP-1, IL-6, IL-10, IL-1β, and TNF-α, while their secretion was more pronounced in the case of TLR7/8 agonist-treated PBMCs. Notably, stimulation with TLR7/8 agonists alone also induced moderate levels of IFN-γ and the IFN-inducible chemokine IP-10. The synergistic effect of synthesized conjugated NOD2/TLR7 agonists on the cytokine producing capacity of PBMCs was convincingly demonstrated by their similar yet robust inflammatory signatures characterized by the production of IL-6, MCP-1, IL-1β, and TNF-α. Importantly, for all conjugated NOD2/TLR7 agonists the synergy extended to IL-12p70, IFN-γ, and IP-10, which ultimately translates to a Th1 polarized response,^37−40^ while the observed increase in the levels of TGF-β1 in conjunction with elevated IL-6 levels can drive the response also toward the Th17 type.^41^ Moreover, conjugated NOD2/TLR7 agonists, albeit to a lesser extent, synergistically augmented the secretion of the Th2 cytokine IL-4, which is conducive to B cell development, in particular with concomitant TNF-α release. In most cases, the concentrations of secreted cytokines following stimulation with the conjugates surpassed those induced by the unconjugated mixture of agonists, indicating that covalent conjugation resulted in enhanced immune cell stimulation. As to the impact of the connecting spacer, no major differences in overall levels of induced cytokines/chemokines have been observed between conjugates featuring flexible aliphatic or flexible PEG-based linkers, although conjugate **24** featuring an aliphatic linker did slightly surpass the PEG-based linker carrying conjugate **23** in terms of IL-12p70, IFN-γ, IL-1β, and TNF-α secretion. Nonetheless, this observation is in line with the report from Ryu et al., who demonstrated no significant linker length effect in conjugated TLR agonists for primary immune cells.^28^ In fact, the only evident exception was seen with conjugate **22** that increased the production of IL-2 and IL-10 to a significantly greater extent than other conjugates while exhibiting lesser capacity to induce IL-4, IL-6, IL-1β, TNF-α, IL-12p70, and IFN-γ. Conversely, a less pronounced effect on overall cytokine secretion was seen with the triazole-based click conjugate **29**. The linkage site on NOD2 agonist obviously also plays an important role given that conjugate **26** where α-COOH of the d-Glu was exploited as the attachment site is far inferior in comparison to its positional isomer **22** based on the overall levels of induced cytokines/chemokines. Importantly, compared to **22**-**24**, conjugates **26** and **29** produced less Th1 cytokines (IL-12p70 and IFN-γ), while conjugate **29** also failed to elicit the release of IL-1β, IL-8, and IL-4 to the extent observed with other conjugates as well as with the mixture of individual ligands (**4**+**5**). Co-treatments with mixtures of NOD2 and imidazoquinoline TLR7/8 agonists demonstrated pronounced synergy as evidenced by the extent of cytokine production. Unfortunately, their conjugated counterparts **32** and **33** were devoid of any potential synergistic effect in this regard (Figure S2). It is worth mentioning that modifications of the C-4 amine group in both resiquimod and aminoresiquimod have previously been shown to be well-tolerated in an *in vivo* setting^42−45^ while the *in vitro* conditions were not conducive to such structural alterations.^33^

**Figure 3:**
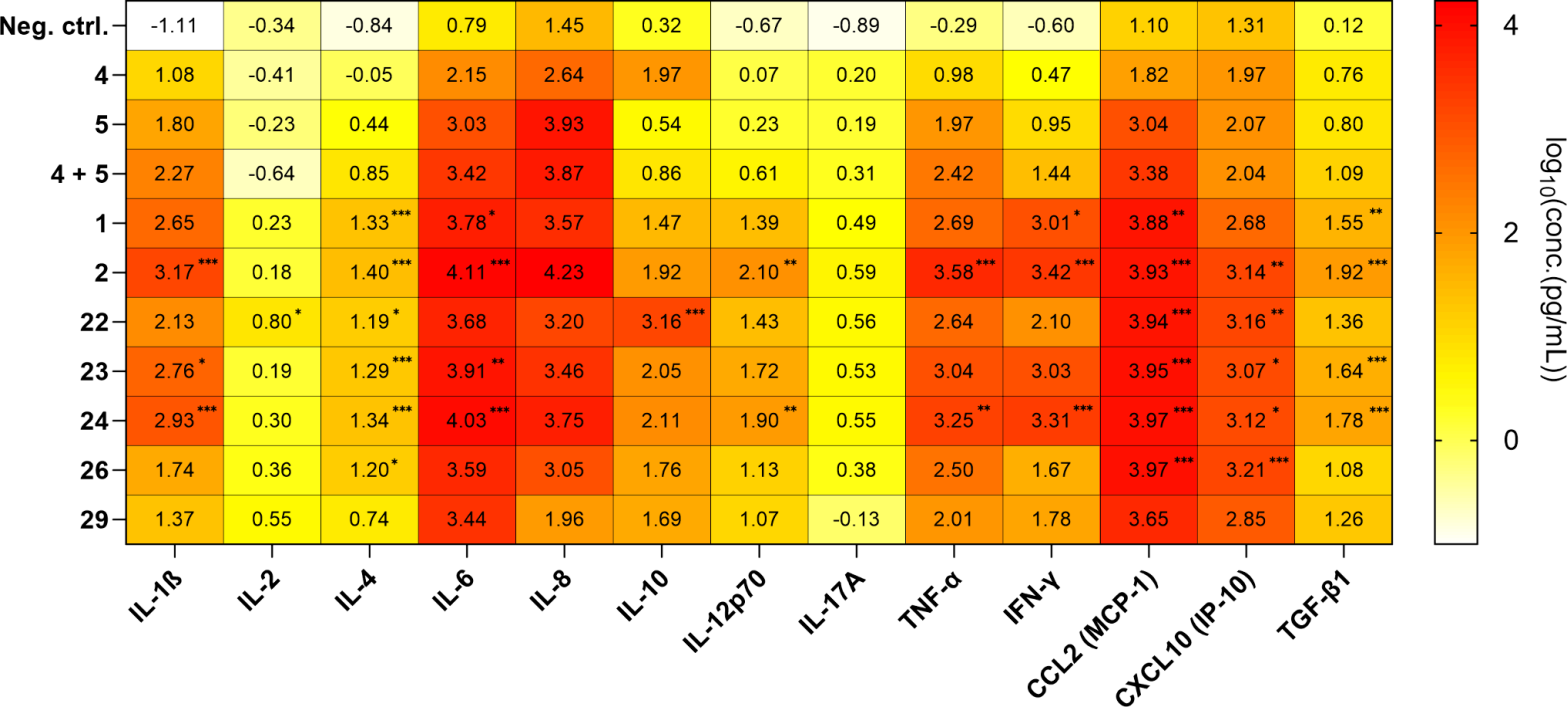
Heatmap of the cytokines logarithmic concentrations measured after 18 h of stimulation of human PBMCs with individual NOD2 and TLR7 agonists, their mixture, and conjugated NOD2/TLR7 agonists (1 μM). The data are mean values of three independent experiments. *, *p* < 0.05, **, *p* < 0.001, ***, *p* < 0.001 versus combination of **4** + **5** (one-way ANOVA *post hoc* Dunnett’s test).

In conclusion, the results presented here shed additional light on the SAR of conjugated NOD2/TLR7 agonists. The immune-stimulating activities of the conjugates were demonstrated through enhanced activation of primary human immune cells, resulting in a potent Th1/Th17-biased cytokine secretion profile. Compound **24** was identified as the most potent immunostimulant of the series. Furthermore, a comparison between the conjugated agonists illustrates how the chemical nature of the linker and the attachment site on the NOD2 agonist can affect both the physicochemical properties (e.g., kinetic solubility) and the immunostimulatory capacity of these conjugates. The γ-carboxylic group of NOD2 agonist emerged as the optimal attachment site for the linker, while the carboxylic group on the TLR7 agonist has been well established to accommodate any substrate. The aromatic amine group of resiquimod and the aliphatic amine group from the aminoresiquimod both proved as unsuitable sites for linker attachment. Importantly, our study highlights how physicochemical limitations can be avoided by the use of an appropriate linker. The results presented here are noteworthy as they provide a roadmap to the design of conjugated innate immune agonists with improved physicochemical properties based on chemical manipulation of the linker portion. Future *in vitro* and *in vivo* studies will assess the utility of conjugated NOD2/TLR7 agonists as vaccine adjuvants and/or immunotherapeutics.

## Supporting Information Available

Supporting Figures; Supporting Tables; Experimental details; Compound characterization (PDF)

## Abbreviations

COMU: 1-[(1-(cyano-2-ethoxy-2-oxoethylideneaminooxy)-dimethylamino-morpholinomethylene)]-methanaminium hexafluorophosphate
DC: dendritic cells
DCM: dichloromethane
DIPEA: *N*,*N*-diisopropylethylamine
HATU: 1-[bis(dimethylamino)methylene]-1*H*-1,2,3-triazolo[4,5-*b*]pyridinium 3-oxide hexafluorophosphate
IFN: interferon
IL: interleukin
IP-10: interferon gamma-induced protein 10
MCP-1: monocyte chemoattractant protein-1
MDP: muramyl dipeptide
NF-κB: nuclear factor κB
NK: natural killer
NOD: nucleotide-binding oligomerization domain
PBMC: peripheral blood mononuclear cell
PEG: polyethylene glycol
PRR: pattern recognition receptor
SAR: structure–activity relationship
SEAP: secreted embryonic alkaline phosphatase
TFA: trifluoroacetic acid
TGF: transforming growth factor
TLR: Toll-like receptor
TNF: tumor necrosis factor
