# Supplementary material for: Linker Chemistry and Connectivity Fine-Tune the Immune Response and Kinetic Solubility of Conjugated NOD2/TLR7 Agonists
Source: Bioconjug Chem. 2024 Oct 10;35(11):1723–31. doi: 10.1021/acs.bioconjchem.4c00321 (PMC11583123; doi:10.1021/acs.bioconjchem.4c00321)
Supplement: Supplementary file 1 — bc4c00321_si_001.pdf [file bc4c00321_si_001.pdf]

SUPPORTING INFORMATION

Linker chemistry and connectivity fine-tune the immune response and kinetic solubility of conjugated NOD2/TLR7 agonists

Špela Janež, Samo Guzelj, Žiga Jakopin*

Faculty of Pharmacy, University of Ljubljana, SI-1000 Ljubljana, Slovenia

*Corresponding Author

Žiga Jakopin

Phone: +386 1 4769 646

Fax: +386 1 4258 031

E-mail: ziga.jakopin@ffa.uni-lj.si

Table of contents

1. Supporting figures 2

2. Synthetic procedures 3

3. Biology 12

4. Representative NMR Spectra....................13

5. Representative UHPLC traces 18

6. Supporting table 21

7. Abbreviations....................22

8. References 22

1. Supporting figures

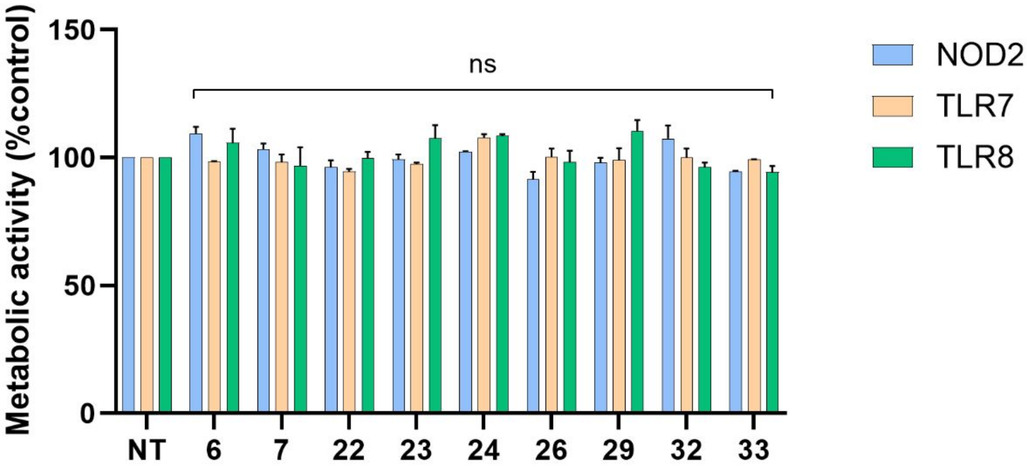

Figure S1. NOD2/TLR7 and NOD2/TLR7/8 conjugates are not cytotoxic towards HEK-Blue cells. Metabolic activities of HEK-Blue NOD2, HEK-Blue TLR7 and HEK-Blue TLR8 were measured after 18 h treatment with the compounds (10 µM). Data are shown relative to the untreated control (0.1% DMSO). Data are means ±SEM of two independent experiments. ns, not significant *versus* NT (one-way ANOVA *post hoc* Dunnett's tests).

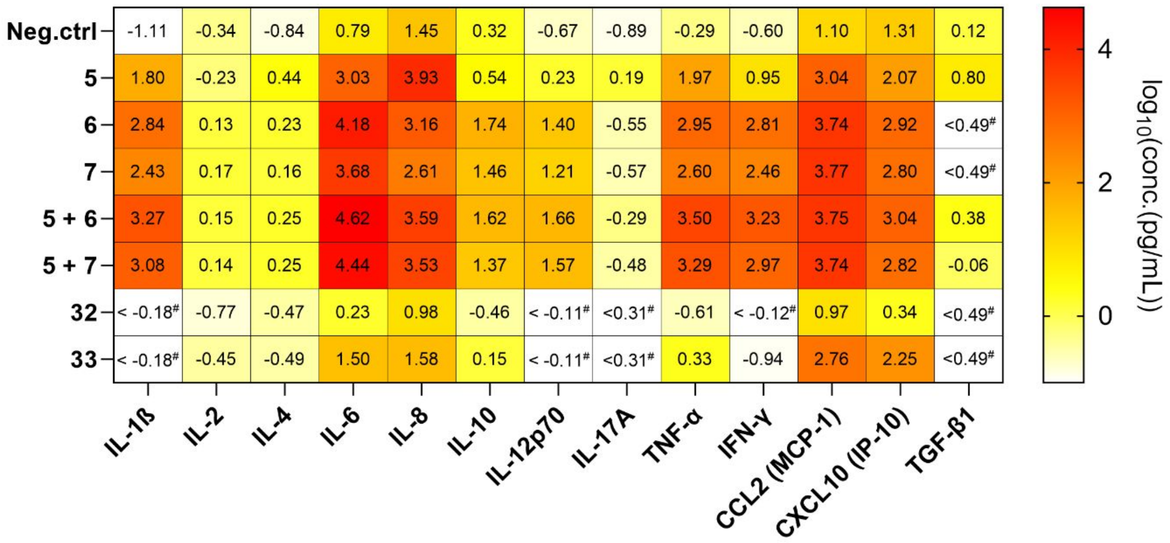

Figure S2. Heatmap of the cytokines logarithmic concentrations measured after 18 hours of stimulation of human PBMCs with the compounds (1 µM). The data are mean values of three independent experiment. #Minimum detectable concentration. Cytokine releases induced by **32** and **33** are not significantly increased versus combinations of **5** + **6** or **5** + **7**, respectively.

2. Synthetic procedures

2.1 Materials

Chemicals were obtained from Sigma-Aldrich (St. Louis, MO, USA), BLD Pharm (Germany), Acros Organics (Geel, Belgium), ABCR (Germany), Enamine (Monmouth Junction, NJ, USA), and Apollo (Stockport, UK), and were used without further purification. Analytical TLC was performed on Merck 60 F254 silica gel plates (0.25 mm), with visualization using ultraviolet light and ninhydrin. Flash column chromatography was carried out on Merck silica gel 60 (particle size 240−400 mesh) and on Biotage Isolera One Flash Chromatograph using Biotage® Sfär C18 D Duo 100 Å 30 µm 30 g. ^{1}H and ^{13}C NMR spectra were recorded at 400 MHz and 100 MHz, respectively, on an Avance III spectrometer (Bruker Corporation, Billerica, MA, USA) in $CDCl_3$ or DMSO-d_6 with tetramethylsilane as the internal standard. Mass spectra were obtained using an Exactive Plus orbitrap mass spectrometer (Thermo Fisher Scientific, Waltham, MA, USA) or on Expression CMS mass spectrometer (Advion Inc., Ithaca, NY, USA). Analytical UHPLC analyses were performed on a Dionex UltiMate 3000 Rapid Separation Binary System (Thermo Fisher Scientific, Waltham, MA, USA) equipped with an autosampler, a binary pump system, a photodiode array detector, a thermostated column compartment, and the Chromeleon Chromatography data system. The column used was Waters Acquity UPLC BEH C18 (1.7 µm, 2.1 × 50 mm), with a flow rate of 0.3 mL/min. The eluent was a mixture of 0.1% TFA in water (A) and acetonitrile (B), with a gradient of (%B): 0–10 min, 5–95%; 10–12 min, 95%; 12–12.5 min, 95-5%. The columns were thermostated at 40 °C. The purity of all biologically tested compounds was >95%.

Compounds **4** and **7** were prepared as described previously.[1,2]

2.2 General synthetic procedures

2.2.1 General procedure A: Boc protection of diamines.

To an ice-chilled stirred solution of diamine (5 eq) in DCM, di-tert-butyl dicarbonate (1 eq) in DCM was added dropwise. The stirring was continued overnight at room temperature, after which DCM was evaporated off in vacuo and saturated $NaHCO_3$ (60 mL) was added. The resulting mixture was extracted with DCM (2 × 50 mL) and the combined organic phases were washed with brine (20 mL). The resulting organic layer was dried over anhydrous Na_2SO_4 and concentrated *in vacuo*.

2.2.2 General procedure B: TFA-mediated acidolysis.

The Boc-protected compound was added to an ice-chilled stirred mixture of TFA and DCM (1:5), and the mixture was allowed to warm to room temperature. After 3 h, the solvent was evaporated off *in vacuo*. The residue was washed three times with diethyl ether.

2.2.3 General procedure C: EDC-Mediated coupling 1.

To an ice-chilled stirring solution of amine (1 eq) in DCM, carboxylic acid (1.2 eq), DIPEA (5 eq), DMAP (catalytic amount), HOBt (1.2 eq) and EDC × HCl (1.2 eq) were added. The mixture was allowed to warm to room temperature and the stirring continued overnight after which DCM (30 mL) was added and washed with 1 M HCl (2 × 50 mL), saturated $NaHCO_3$ (2 × 50 mL), brine (30 mL), dried over anhydrous Na_2SO_4, concentrated *in vacuo.*

2.2.4 General procedure D: COMU-Mediated coupling 2.

To an ice-chilled stirred solution of amine (3.33 eq) and carboxylic acid (1 eq) in DMSO/DMF, DIPEA (5.33 eq) and COMU (2.66 eq) were added. The mixture was allowed to warm to room temperature

and the stirring continued overnight, after which it was poured to a flask containing $NaHCO_3$ (75 mL) and ethyl acetate (50 mL). Ethyl acetate was evaporated off *in vacuo* and the resulting precipitate was chilled on ice, filtrated, and washed with cold water and ether.

2.2.5 General procedure E: COMU-Mediated coupling 3.

To an ice-chilled stirred solution of the amine (1.2 eq) and carboxylic acid (1 eq) in DMF, DIPEA (3.5 eq) and COMU (1.3 eq) were added. The mixture was allowed to warm to room temperature and the stirring continued overnight, after which 1M HCl was added (20 mL) and stirred for 10 minutes. The resulting mixture was extracted with DCM/isopropyl alcohol (3:1) (3 × 20 mL). Combined organic phases were washed with saturated $NaHCO_3$ (2 × 30 mL), brine (30 mL), dried over anhydrous Na_2SO_4 and concentrated *in vacuo*.

Scheme S1: Synthesis of linkers **8**-**13**.

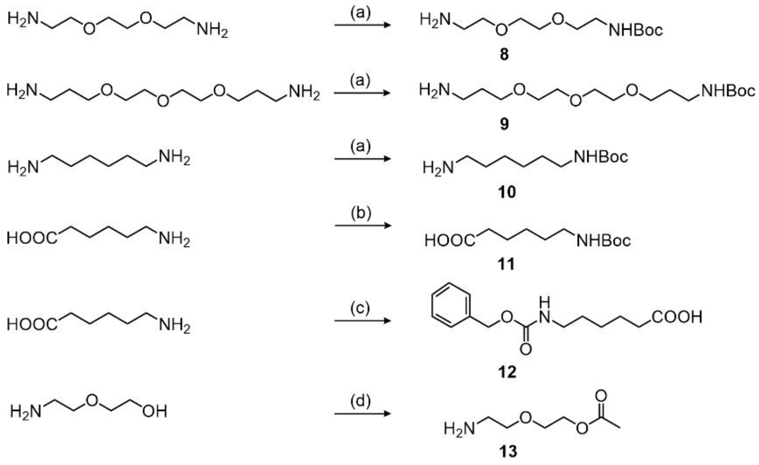

Reagents and conditions: (a) Boc_2O, DCM, rt.; (b) Boc_2O, NaOH/H_2O, dioxane, rt.; (c) benzyl chloroformate, K_2CO_3, THF, rt.; (d) CH_3COOH, CH_3COCl, 2 h, 0°C.

2.3 Characterization of compounds

2.3.1 tert-butyl (2-(2-(2-aminoethoxy)ethoxy)ethyl)carbamate (8)

Synthesized from 1,2-bis(2-aminoethoxy)ethane) (14.82 g, 100 mmol) using General procedure A. Colorless oil (3.24 g, 65%). ^{1}H NMR (400 MHz, DMSO-d_6) δ 6.92 – 6.64 (m, 1H), 3.49 (d, J = 7.2 Hz, 4H), 3.43 – 3.27 (m, 4H), 3.06 (q, J = 6.0 Hz, 2H), 2.64 (t, J = 5.8 Hz, 2H), 1.37 (s, 9H).

2.3.2 tert-butyl (3-(2-(2-(3-aminopropoxy)ethoxy)ethoxy)propyl)carbamate (9)

Synthesized from diethylene glycol bis(3-aminopropyl)ether) (15.00 g, 69 mmol) using General procedure A. Colorless oil (3.32 g, 74%). ^{1}H NMR (400 MHz, DMSO-d_6) δ 6.87 – 6.73 (m, 1H), 3.56 – 3.32 (m, 11H), 3.02 – 2.86 (m, 2H), 2.57 (t, J = 6.8 Hz, 2H), 1.62 – 1.47 (m, 4H), 1.37 (s, 9H).

2.3.3 tert-butyl (6-aminohexyl)carbamate (10)

Synthesized from 1,6-diaminohexane (10.00 g, 86 mmol) using General procedure A. White solid (2.47 g, 67%). ^{1}H NMR (400 MHz, DMSO-d_6) ^{1}H NMR (400 MHz, DMSO-d_6) δ 6.77 (t, J = 5.7 Hz, 1H), 2.95 – 2.80 (m, 2H), 1.46 – 1.13 (m, 19H). (Signals for 2 protons are under solvent peak)

2.3.4 6-((tert-butoxycarbonyl)amino)hexanoic acid (11)

6-aminohexanoic acid (3.00 g, 22.9 mmol) was dissolved in water (8 mL) and 1 M NaOH (40 mL), while di-tert-butyl dicarbonate (6.49 g, 29.7 mmol) was dissolved in dioxane (15 mL). The reaction mixtures were combined on ice and stirred at room temperature overnight. Subsequently 1 M NaOH (4 mL) was added to increase the pH to 10. Dioxane was evaporated off *in vacuo* and the residual was washed with ether (20 mL). Water phase was acidified with 1 M HCl to pH 2 and extracted with ethyl acetate (3 × 70 mL). Combined organic layers were dried over Na_2SO_4 and concentrated *in vacuo* to produce compound **11** as a colorless oil (4.55 g, 86%). ^{1}H NMR (400 MHz, DMSO-d_6) δ 11.98 (s, 1H), 6.77 (t, *J* = 5.8 Hz, 1H), 3.57 (s, 1H), 2.88 (q, *J* = 6.9 Hz, 2H), 2.18 (t, *J* = 7.4 Hz, 2H), 1.55 – 1.41 (m, 2H), 1.41 – 1.28 (m, 11H), 1.28 – 1.13 (m, 3H).

2.3.5 6-(((Benzyloxy)carbonyl)amino)hexanoic acid (12)

To an ice-chilled stirring suspension of 6-aminohexanoic acid (1.312 g, 10 mmol) and K_2CO_3 (2.764 g, 20 mmol) in THF (20 mL), benzyl chloroformate (2.133 mL, 15 mmol) was added dropwise. The resulting suspension was stirred at room temperature for 20 h. Subsequently, ethyl acetate (20 mL) and water (30 mL) were added. Water phase was acidified with 4 M HCl to pH 2 and extracted with ethyl acetate (15 mL × 2). Combined organic phases were washed with brine (15 mL), dried over anhydrous Na_2SO_4, and concentrated *in vacuo* to give compound **12** as a white solid (1.71 g, 65%).^{1}H NMR (400 MHz, Chloroform-*d*) δ 7.43 – 7.28 (m, 5H), 5.09 (s, 2H), 4.86 – 4.74 (m, 1H), 3.27 – 3.08 (m, 2H), 2.41 – 2.30 (m, 2H), 1.71 – 1.30 (m, 7H).

2.3.6 2-(2-Aminoethoxy)ethyl acetate (13)

To an ice-chilled stirring solution of 2-(2-aminoethoxy)ethan-1-ol (1.577 g, 15 mmol) in acetic acid (4 mL), acetyl chloride (4.3 mL, 60 mmol) was added dropwise. The resulting solution was stirred on ice for 2 h, after which it was concentrated *in vacuo*. The precipitate was washed with ether three times and filtrated, to give compound **13** as a yellow solid (2.0 g, 90%). ^{1}H NMR (400 MHz, DMSO-d_6) δ 8.07 (s, 3H), 4.21 – 4.08 (m, 2H), 3.71 – 3.55 (m, 4H), 2.95 (d, *J* = 5.5 Hz, 2H), 2.03 (s, 3H).

Scheme S2: Synthesis of NOD2 agonist **5a**.

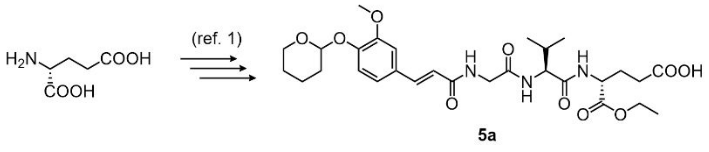

Reagents and conditions: Used as reported in [1].

Scheme S3: Synthesis of NOD2 agonist **5b**.

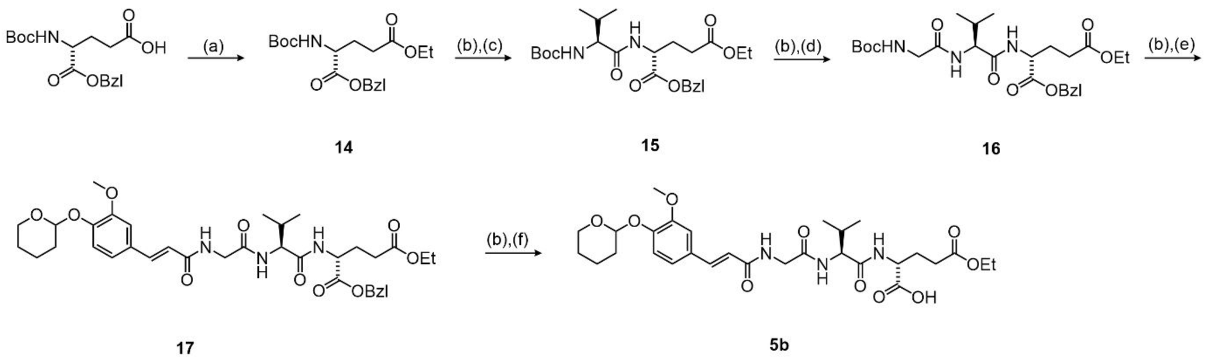

Reagents and conditions: (a) EtOH, EDC × HCl, HOBt, DMAP, DCM, rt.; (b) TFA/DCM (5:1); (c) Boc-L-val, EDC × HCl, HOBt, DMAP, DIPEA, DCM, rt.; (d) Boc-glycine, EDC × HCl, HOBt, DMAP, DIPEA, DCM, rt.; (e) (E)-3-(3-Methoxy-4-((tetrahydro-2H-pyran-2-yl)oxy)phenyl) acrylic acid, EDC × HCl, HOBt, DMAP, DIPEA, DMF, rt; (f) $Pd(OAc)_2$, Et_3SiH, TEA, DCM, rt.

2.3.7 1-Benzyl 5-ethyl (tert-butoxycarbonyl)-D-glutamate (14)

To an ice-chilled stirring solution of tert-butoxycarbonyl-D-glutamic acid 1-benzyl ester (3.374 g, 10 mmol) in DCM (50 mL), absolute ethanol (5 mL), DMAP (0.120 g, 0.1 mmol), HOBt (2.027 g, 15 mmol) and EDC × HCl (2.876 g, 15 mmol) were added. The mixture was allowed to warm to room temperature and the stirring continued overnight after which DCM (50 mL) was added and washed with 1 M HCl (2 × 50 mL), saturated $NaHCO_3$ (2 × 50 mL), brine (30 mL), dried over anhydrous Na_2SO_4, concentrated *in vacuo* and two times coevaporated with ether to give compound **14** as a yellow oil (3.61 g, 99%). ^{1}H NMR (400 MHz, DMSO-d_6) δ 7.41 – 7.30 (m, 6H), 5.20 – 5.03 (m, 2H), 4.10 – 3.97 (m, 3H), 2.44 – 2.28 (m, 2H), 2.05 – 1.89 (m, 1H), 1.89 – 1.72 (m, 1H), 1.37 (s, 9H), 1.16 (t, *J* = 7.1 Hz, 3H).

2.3.8 1-Benzyl 5-ethyl (tert-butoxycarbonyl)-L-valyl-D-glutamate (15)

Compound **14** (3.515 g, 9.62 mmol) was deprotected using General procedure B and coupled to Boc-L-valine (2.507 g, 11.54 mmol) following General procedure C to give compound **15** as a yellow oil (4.37 g, 98%). ^{1}H NMR (400 MHz, DMSO-d_6) δ 8.30 (d, *J* = 7.6 Hz, 1H), 7.42 – 7.27 (m, 5H), 6.64 (d, *J* = 8.9 Hz, 1H), 5.16 – 5.07 (m, 2H), 4.38 – 4.22 (m, 1H), 4.10 – 3.92 (m, 2H), 3.87 – 3.76 (m, 1H), 2.42 – 2.25 (m, 2H), 2.09 – 1.95 (m, 1H), 1.95 – 1.74 (m, 2H), 1.38 (s, 9H), 1.16 (t, *J* = 7.1 Hz, 3H), 0.88 – 0.71 (m, 6H).

2.3.9 1-Benzyl 5-ethyl (tert-butoxycarbonyl)glycyl-L-valyl-D-glutamate (16)

Compound **15** (4.238 g, 9.12 mmol) was deprotected using General procedure B and coupled to Boc-glycine (1.918 g, 10.95 mmol) following General procedure C to give compound **16** as a yellow solid (3.67 g, 77%). ^{1}H NMR (400 MHz, DMSO-d_6) δ 8.49 (d, *J* = 7.6 Hz, 1H), 7.69 – 7.58 (m, 1H), 7.44 – 7.27 (m, 5H), 7.04 (t, *J* = 6.0 Hz, 1H), 5.16 – 5.07 (m, 2H), 4.40 – 4.13 (m, 2H), 4.09 – 3.97 (m, 2H), 3.66 – 3.44 (m, 2H), 2.42 – 2.27 (m, 2H), 2.10 – 1.75 (m, 3H), 1.38 (s, 9H), 1.15 (t, *J* = 7.1 Hz, 3H), 0.92 – 0.73 (m, 6H).

2.3.10 1-Benzyl 5-ethyl ((E)-3-(3-methoxy-4-((tetrahydro-2H-pyran-2-yl)oxy)phenyl)acryloyl)glycyl-L-valyl-D-glutamate (17)

Compound **16** (0.500 g, 0.959 mmol) was deprotected using General procedure B and coupled to (E)-3-(3-Methoxy-4-((tetrahydro-2H-pyran-2-yl)oxy)phenyl) acrylic acid (0.294 g, 1.055 mmol) following General procedure C (DMF was used as a solvent, and ethyl acetate in extraction). The crude product

was purified using column chromatography (MeOH/DCM 1:25) to give compound **17** as an off-white solid (0.25 g, 38%). ^{1}H NMR (400 MHz, DMSO-d_6) δ 8.45 (d, J = 7.5 Hz, 1H), 8.25 (t, J = 5.8 Hz, 1H), 7.94 (d, J = 9.0 Hz, 1H), 7.42 – 7.30 (m, 7H), 7.24 – 7.19 (m, 1H), 7.11 – 7.07 (m, 1H), 6.66 (d, J = 15.8 Hz, 1H), 5.46 (t, J = 3.3 Hz, 1H), 5.17 – 5.08 (m, 2H), 4.40 – 4.32 (m, 1H), 4.32 – 4.21 (m, 1H), 4.07 – 3.97 (m, 2H), 3.91 – 3.87 (m, 2H), 3.82 (s, 3H), 3.38 (q, J = 7.0 Hz, 2H), 2.38 – 2.32 (m, 2H), 2.05 – 1.70 (m, 6H), 1.68 – 1.51 (m, 3H), 1.17 – 1.11 (m, 3H), 0.87 – 0.80 (m, 6H).

2.3.11 (2R)-5-Ethoxy-2-((2S)-2-(2-((E)-3-(3-methoxy-4-((tetrahydro-2H-pyran-2-yl)oxy)phenyl)acrylamido)acetamido)-3-methylbutanamido)-5-oxopentanoic acid (5b)

To a solution of palladium (II) acetate (4 mg, 0.016 mmol) in dry DCM (5 mL), Et_3N (87 µL, 0.624 mmol) and triethylsilane (102 µL, 0.640 mmol) were added. The resulting black solution was stirred at room temperature for 15 min, after which a solution of compound **17** (218 mg, 0.320 mmol) in dry DCM (7 mL) was added. The resulting mixture was stirred under argon atmosphere at room temperature for 18 h, after which the catalyst was removed by filtration and washed with DCM (20 mL). The product was extracted with water (3 × 30 mL). The combined aqueous phases were acidified with 1 M HCl and extracted with DCM (3 × 30 mL). The combined organic phases were washed with water (50 mL), dried over anhydrous Na_2SO_4 and concentrated *in vacuo* to give compound **5a** as a white solid (140 mg, 75%). ^{1}H NMR (400 MHz, DMSO-d_6) δ 12.68 (s, 1H), 8.40 – 8.14 (m, 2H), 7.90 (d, J = 9.0 Hz, 1H), 7.37 (d, J = 15.7 Hz, 1H), 7.21 (s, 1H), 7.16 – 6.96 (m, 2H), 6.65 (d, J = 15.8 Hz, 1H), 5.46 (t, J = 3.3 Hz, 1H), 4.35 – 4.14 (m, 2H), 4.14 – 3.96 (m, 2H), 3.89 (d, J = 5.8 Hz, 2H), 3.82 (s, 3H), 3.62 – 3.48 (m, 1H), 2.38 – 2.21 (m, 3H), 2.06 – 1.67 (m, 7H), 1.67 – 1.39 (m, 3H), 1.19 – 1.12 (m, 3H), 0.87 – 0.79 (m, 6H).

2.3.12 tert-butyl (2-(2-(2-(4-((6-amino-2-butoxy-8-hydroxy-9H-purin-9-yl)methyl)benzamido)ethoxy)ethoxy)ethyl)carbamate (18)

Synthesized from **8** (0.162 g, 0.652 mmol) and **4** (0.070 g, 0.196 mmol) in DMSO (3.5 mL) using General procedure D. White solid (93 mg, 80%). ^{1}H NMR (400 MHz, DMSO-d_6) δ 10.14 (s, 1H), 8.48 (t, J = 5.6 Hz, 1H), 7.84 – 7.76 (m, 2H), 7.34 (d, J = 8.2 Hz, 2H), 6.84 – 6.68 (m, 1H), 6.52 (s, 2H), 4.90 (s, 2H), 4.12 (t, J = 6.6 Hz, 2H), 3.61 – 3.45 (m, 8H), 3.45 – 3.38 (m, 2H), 3.07 – 3.02 (m, 2H), 1.69 – 1.53 (m, 2H), 1.37 – 1.29 (m, 11H), 0.89 (t, J = 7.4 Hz, 3H).

2.3.13 tert-butyl (1-(4-((6-amino-2-butoxy-8-hydroxy-9H-purin-9-yl)methyl)phenyl)-1-oxo-6,9,12-trioxa-2-azapentadecan-15-yl)carbamate (19)

Synthesized from **9** (0.746 g, 2.329 mmol) and **4** (0.250 g, 0.670 mmol) in DMSO (8 mL) using General procedure D. White solid (362 mg, 82%). ^{1}H NMR (400 MHz, DMSO-d_6) δ 8.39 (t, J = 5.7 Hz, 1H), 7.77 (d, J = 8.0 Hz, 2H), 7.34 (d, J = 7.8 Hz, 2H), 6.92 – 6.63 (m, 1H), 6.48 (s, 2H), 4.90 (s, 2H), 4.13 (t, J = 6.7 Hz, 2H), 3.62 – 3.40 (m, 15H), 2.99 – 2.91 (m, 4H), 1.79 – 1.67 (m, 2H), 1.65 – 1.54 (m, 5H), 1.42 – 1.30 (m, 15H), 0.90 (t, J = 7.4 Hz, 3H).

2.3.14 tert-butyl (6-(4-((6-amino-2-butoxy-8-hydroxy-9H-purin-9-yl)methyl)benzamido)hexyl)carbamate (20)

Synthesized from **10** (0.500 g, 2.329 mmol) and **4** (0.250 g, 0.670 mmol) in DMSO (8 mL) using General procedure D. White solid (372 mg, 100%). ^{1}H NMR (400 MHz, DMSO-d_6) δ 9.98 (s, 1H), 8.39 (t, J = 5.4 Hz, 1H), 7.77 (d, J = 8.0 Hz, 2H), 7.34 (d, J = 8.0 Hz, 2H), 6.87 – 6.64 (m, 2H), 6.48 (s, 2H), 4.90 (s, 2H), 4.13 (t, J = 6.7 Hz, 2H), 2.96 – 2.86 (m, 6H), 1.67 – 1.57 (m, 2H), 1.57 – 1.43 (m, 3H), 1.37 – 1.33 (m, 11H), 1.30 – 1.15 (m, 7H), 0.90 (t, J = 7.4 Hz, 3H).

2.3.15 2-(2-(4-((6-amino-2-butoxy-8-hydroxy-9H-purin-9-yl)methyl)benzamido)ethoxy)ethyl acetate (21)

To a suspension of **4** (0.150 g, 0.420 mmol) in DMSO (7 mL), **10** (0.260 g, 1.26 mmol) dissolved in DCM (2 mL), DIPEA (0.585 mL, 3.36 mmol) and COMU (0.450 g, 1.05 mmol) were added. The reaction mixture was stirred at room temperature for 3 h, after which it was poured to a flask containing ethyl acetate (40 mL) and put on ice for 1 h. Subsequently 1 M $NaHCO_3$ (30 mL) was added and the organic solvent was evaporated *in vacuo* and the resulting precipitate was chilled on ice for 1 h after which it was filtrated and washed with cold water to give compound **21** as a white solid (115 mg, 56%).

2.3.16 Ethyl (R)-1-(4-((6-amino-2-butoxy-8-hydroxy-9H-purin-9-yl)methyl)phenyl)-15-((S)-2-(2-((E)-3-(4-hydroxy-3-methoxyphenyl)acrylamido)acetamido)-3-methylbutanamido)-1,12-dioxo-5,8-dioxa-2,11-diazahexadecan-16-oate (22)

Compound **18** (0.049 g, 0.081 mmol) was deprotected using General procedure B and coupled to **5a** (0.040 g, 0.068 mmol) using General procedure E. The crude product was purified by Isolera One flash chromatography (acetonitrile/0.1% TFA 20% → 100%) to obtain a compound **22** as a pale-yellow solid (6 mg, 9%). ^{1}H NMR (400 MHz, DMSO-d_6) δ 8.42 (t, *J* = 5.6 Hz, 1H), 8.34 (d, *J* = 7.2 Hz, 1H), 8.13 (t, *J* = 5.8 Hz, 1H), 7.86 – 7.76 (m, 2H), 7.76 – 7.65 (m, 2H), 7.33 – 7.20 (m, 3H), 7.09 – 7.04 (m, 1H), 6.92 (dd, *J* = 8.3, 1.9 Hz, 1H), 6.76 – 6.65 (m, 1H), 6.58 – 6.42 (m, 3H), 4.83 (s, 2H), 4.22 – 3.96 (m, 6H), 3.83 (d, *J* = 5.7 Hz, 2H), 3.73 (s, 3H), 3.46 – 3.38 (m, 7H), 3.34 – 3.27 (m, 5H), 3.14 – 3.07 (m, 3H), 2.11 – 2.02 (m, 2H), 1.98 – 1.83 (m, 2H), 1.80 – 1.68 (m, 1H), 1.63 – 1.44 (m, 2H), 1.35 – 1.20 (m, 2H), 1.16 – 1.04 (m, 3H), 0.85 – 0.72 (m, 9H). ^{13}C NMR (100 MHz, DMSO) δ 172.23, 171.68, 171.48, 169.51, 166.46, 166.35, 160.40, 149.63, 148.48, 148.45, 140.86, 140.07, 133.94, 127.85, 127.63, 122.27, 118.51, 116.20, 111.24, 69.99, 69.94, 69.54, 69.36, 66.24, 62.48, 60.93, 57.84, 55.93, 52.19, 42.74, 42.55, 40.88, 40.65, 40.59, 40.38, 40.18, 39.97, 39.76, 39.55, 39.34, 38.98, 31.80, 31.25, 31.06, 27.14, 25.95, 19.65, 19.21, 18.20, 14.49, 14.18. HRMS *m/z* calculated for $C_{47}H_{65}O_{13}N_{10}$: 977.4727 (M + H)$^+$, found 977.4715.

2.3.17 Ethyl (R)-1-(4-((6-amino-2-butoxy-8-hydroxy-9H-purin-9-yl)methyl)phenyl)-20-((S)-2-(2-((E)-3-(4-hydroxy-3-methoxyphenyl)acrylamido)acetamido)-3-methylbutanamido)-1,17-dioxo-6,9,12-trioxa-2,16-diazahenicosan-21-oate (23)

Compound **19** (54 mg, 0.081 mmol) was deprotected using General procedure B and coupled to **5a** (40 mg, 0.068 mmol) using General procedure E. The crude product was purified by Isolera One flash chromatography (acetonitrile/0.1% TFA 20% → 100%) to obtain a compound **23** as a pale-yellow solid (4 mg, 5%). ^{1}H NMR (400 MHz, DMSO-d_6) δ 8.46 – 8.34 (m, 2H), 8.18 (t, *J* = 5.8 Hz, 1H), 7.91 – 7.83 (m, 1H), 7.82 – 7.70 (m, 3H), 7.37 – 7.26 (m, 2H), 7.14 – 7.02 (m, 1H), 6.94 (dd, *J* = 8.2, 2.0 Hz, 1H), 6.82 – 6.56 (m, 3H), 6.56 – 6.41 (m, 1H), 4.90 (s, 2H), 4.32 – 3.99 (m, 6H), 3.99 – 3.82 (m, 2H), 3.77 (s, 3H), 3.67 – 3.13 (m, 10H), 3.05 (q, *J* = 6.4 Hz, 3H), 2.15 – 2.05 (m, 2H), 2.07 – 1.88 (m, 3H), 1.87 – 1.68 (m, 3H), 1.67 – 1.50 (m, 4H), 1.44 – 1.30 (m, 2H), 1.23 – 1.11 (m, 3H), 0.96 – 0.74 (m, 9H). HRMS *m/z* calculated for $C_{51}H_{73}O_{14}N_{10}$: 1049.5302 (M + H)$^+$, found 1049.5280.

2.3.18 Ethyl N^5-(6-(4-((6-amino-2-butoxy-8-hydroxy-9H-purin-9-yl)methyl)benzamido)hexyl)-N^2-((E)-3-(4-hydroxy-3-methoxyphenyl)acryloyl)glycyl-L-valyl-D-glutaminate (24)

Compound **20** (46 mg, 0.081 mmol) was deprotected using General procedure C and coupled to **5a** (40 mg, 0.068 mmol) using General procedure D. The crude product was purified by Isolera One flash chromatography (acetonitrile/0.1% TFA 20% → 100%) to obtain a compound **24** as a pale-yellow solid (3 mg, 6%). ^{1}H NMR (400 MHz, DMSO-d_6) δ 10.06 (s, 1H), 9.47 (d, *J* = 7.8 Hz, 1H), 8.50 – 8.32 (m, 2H),

8.31 – 8.14 (m, 1H), 7.93 – 7.85 (m, 1H), 7.81 – 7.75 (m, 2H), 7.34 (dd, *J* = 11.9, 3.7 Hz, 2H), 7.16 (d, *J* = 2.0 Hz, 1H), 7.01 (d, *J* = 8.1 Hz, 1H), 6.80 (dd, *J* = 8.1, 2.3 Hz, 1H), 6.64 – 6.44 (m, 2H), 4.90 (s, 1H), 4.35 – 3.97 (m, 6H), 3.90 (d, *J* = 5.7 Hz, 2H), 3.83 – 3.76 (m, 3H), 3.58 – 3.55 (m, 2H), 3.09 – 2.87 (m, 8H), 2.75 (s, 4H), 2.17 – 2.05 (m, 2H), 2.03 – 1.90 (m, 2H), 1.86 – 1.74 (m, 1H), 1.65 – 1.57 (m, 1H), 1.54 – 1.45 (m, 2H), 1.42 – 1.21 (m, 9H), 1.20 – 1.14 (m, 3H), 0.95 – 0.73 (m, 9H). HRMS *m/z* calculated for $C_{47}H_{65}O_{11}N_{10}$: 945.4829 $(M + H)^+$, found 945.4813.

2.3.19 Ethyl (R)-1-(4-((6-amino-2-butoxy-8-hydroxy-9H-purin-9-yl)methyl)phenyl)-13-((S)-2-(2-((E)-3-(4-hydroxy-3-methoxyphenyl)acrylamido)acetamido)-3-methylbutanamido)-1,12-dioxo-5,8-dioxa-2,11-diazahexadecan-16-oate (26)

Compound **18** (0.491 g, 0.835 mmol) was deprotected using General procedure B and coupled to **5b** (0.080 g, 0.136 mmol) using DMF (3 mL), DIPEA (71 µL, 0.408 mmol) and COMU (0.064 g, 0.150 mmol) and stirred on room temperature overnight. Subsequently 1 M HCl (10 mL) was added and stirred for 10 minutes. The resulting mixture was extracted with DCM/isopropyl alcohol (3:1) (3 × 30 mL). Combined organic phases were washed with saturated $NaHCO_3$ (3 × 40 mL), brine (40 mL), dried over anhydrous Na_2SO_4 and concentrated *in vacuo*. The crude product was purified using column chromatography (MeOH/DCM 1:9) to give compound **26** as an off-white solid (10 mg, 7,5%). ^1H NMR (400 MHz, DMSO-d_6) δ 10.00 (s, 1H), 9.46 (s, 1H), 8.48 (t, *J* = 5.5 Hz, 1H), 8.29 (d, *J* = 8.1 Hz, 1H), 8.17 (t, *J* = 5.8 Hz, 1H), 8.03 – 7.88 (m, 2H), 7.86 – 7.74 (m, 2H), 7.47 – 7.28 (m, 3H), 7.18 – 7.11 (m, 1H), 7.00 (dd, *J* = 8.1, 1.9 Hz, 1H), 6.79 (d, *J* = 8.2 Hz, 1H), 6.63 – 6.38 (m, 3H), 4.90 (s, 2H), 4.32 – 4.08 (m, 4H), 4.08 – 3.96 (m, 2H), 3.88 (d, *J* = 5.7 Hz, 2H), 3.80 (s, 3H), 3.75 – 3.70 (m, 1H), 3.57 – 3.44 (m, 7H), 3.29 – 3.09 (m, 2H), 2.35 – 2.18 (m, 2H), 2.05 – 1.83 (m, 2H), 1.85 – 1.66 (m, 1H), 1.66 – 1.54 (m, 2H), 1.43 – 1.28 (m, 2H), 1.21 – 1.06 (m, 3H), 1.00 – 0.69 (m, 9H). ^{13}C NMR (100 MHz, DMSO) δ 172.67, 171.44, 171.41, 169.71, 166.45, 166.20, 160.57, 152.69, 149.60, 148.80, 148.27, 140.67, 139.91, 133.98, 127.87, 127.66, 126.79, 122.04, 118.97, 116.11, 111.33, 98.73, 69.99, 69.94, 69.36, 69.24, 66.31, 60.33, 58.51, 55.97, 52.14, 42.57, 40.65, 40.60, 40.39, 40.18, 39.97, 39.76, 39.55, 39.35, 39.00, 31.03, 30.85, 30.46, 27.58, 19.57, 19.20, 18.65, 14.51, 14.17. HRMS *m/z* calculated for $C_{47}H_{65}O_{13}N_{10}$: 977.4727 $(M + H)^+$, found 977.4711.

2.3.20 4-((6-Amino-2-butoxy-8-hydroxy-9H-purin-9-yl)methyl)-N-(prop-2-yn-1-yl)benzamide (27)

To a solution of **4** (0.107 mg, 0.30 mmol) in DMSO (3 mL), DIPEA (0.157 µL, 0.90 mmol), 2-(2-(2-(2-azidoethoxy)ethoxy)ethoxy)ethan-1-amine (0.161 g, 0.60 mmol), and COMU (0.257 mg, 0.60 mmol) were added and the resulting mixture was stirred for 1.5 h on room temperature. Subsequently ethyl acetate (20 mL) and 0.7 M $NaHCO_3$ (15 mL) were added and ethyl acetate was evaporated off *in vacuo*. The resulting precipitate was chilled on ice, filtrated and washed with cold water and ether to give compound **27** as a brownish oil (95 mg, 57%). ^1H NMR (400 MHz, DMSO-d_6) δ 9.98 (s, 1H), 8.47 (t, *J* = 5.6 Hz, 1H), 7.79 (d, *J* = 7.9 Hz, 2H), 7.34 (d, *J* = 7.9 Hz, 2H), 6.47 (s, 1H), 4.90 (s, 2H), 4.37 – 3.84 (m, 2H), 3.66 – 3.38 (m, 16H), 1.86 – 1.46 (m, 2H), 1.46 – 1.21 (m, 2H), 1.00 – 0.68 (m, 3H).

2.3.21 Ethyl N^2-((E)-3-(4-hydroxy-3-methoxyphenyl)acryloyl)glycyl-L-valyl-N^5-(prop-2-yn-1-yl)-D-glutaminate (28)

To an ice chilled solution of **5a** (0.105 g, 0.177 mmol) in dry DMF (2.5 mL), DIPEA (93 µL, 0.532 mmol), propargylamine (14 µL, 0.213 mmol), and HATU (0.081 g, 0.213 mmol) were added. The reaction mixture was stirred for 2.5 h on room temperature. 1 M HCl (20 mL) was added to the solution. After stirring for 15 minutes, ethyl acetate (40 mL) was added. The organic phase was washed with 1 M HCl (20 mL), saturated $NaHCO_3$ (2 × 20, mL), and brine (20 mL), dried over anhydrous Na_2SO_4 and

concentrated *in vacuo to* produce a pale-yellow solid (46 mg, 48%). ^{1}H NMR (400 MHz, DMSO-d_6) δ 9.45 (s, 1H), 8.40 (d, *J* = 7.2 Hz, 1H), 8.29 (t, *J* = 5.5 Hz, 1H), 8.20 (t, *J* = 5.8 Hz, 1H), 7.87 (d, *J* = 9.2 Hz, 1H), 7.39 – 7.31 (m, 1H), 7.19 – 7.11 (m, 1H), 7.01 (dd, *J* = 8.2, 2.1 Hz, 1H), 6.83 – 6.75 (m, 1H), 6.60 – 6.52 (m, 1H), 4.34 – 4.14 (m, 2H), 4.11 – 4.02 (m, 2H), 3.89 (d, *J* = 5.7 Hz, 1H), 3.86 – 3.71 (m, 5H), 3.09 (t, *J* = 2.5 Hz, 1H), 2.24 – 2.09 (m, 2H), 1.99 – 1.88 (m, 2H), 1.86 – 1.73 (m, 1H), 1.21 – 1.15 (m, 3H), 0.89 – 0.77 (m, 6H).

2.3.22 Ethyl N^5-((1-(1-(4-((6-amino-2-butoxy-8-hydroxy-9H-purin-9-yl)methyl)phenyl)-1-oxo-5,8,11-trioxa-2-azatridecan-13-yl)-1H-1,2,3-triazol-4-yl)methyl)-N^2-((E)-3-(4-hydroxy-3-methoxyphenyl)acryloyl)glycyl-L-valyl-D-glutaminate (29)

To a reaction mixture of **27** (0.052 g, 0.093 mmol) and **28** (0.042 mg, 0.077 mmol) in DMF (1.5 mL), 0.5 M $CuSO_4 \times H_2O$ (77 µL, 0.039 mmol) and 1 M Na-ascorbate (193 µL, 0.193 mmol) were added and stirred overnight at room temperature. Reaction mixture was purified using Isolera One flash chromatography (acetonitrile/0.1% TFA 20% → 100%) to obtain a compound **29** as a white solid (8 mg, 9%). ^{1}H NMR (400 MHz, DMSO-d_6) δ 9.98 (s, 1H), 9.45 (s, 1H), 8.62 – 8.28 (m, 3H), 8.28 – 8.13 (m, 1H), 7.97 – 7.73 (m, 4H), 7.48 – 7.29 (m, 3H), 7.15 (s, 1H), 7.04 – 6.98 (m, 1H), 6.86 – 6.74 (m, 1H), 6.64 – 6.39 (m, 2H), 4.90 (s, 2H), 4.53 – 4.41 (m, 2H), 4.34 – 4.03 (m, 9H), 3.95 – 3.67 (m, 9H), 3.62 – 3.45 (m, 14H), 2.24 – 2.06 (m, 2H), 2.06 – 1.89 (m, 2H), 1.89 – 1.71 (m, 1H), 1.69 – 1.49 (m, 2H), 1.43 – 1.26 (m, 2H), 1.25 – 1.05 (m, 4H), 1.04 – 0.69 (m, 9H). HRMS *m/z* calculated for $C_{52}H_{72}O_{14}N_{13}$: 1102.5316 (M + H)$^+$, found 1102.5295.

2.3.23 tert-butyl (6-((2-(ethoxymethyl)-1-(2-hydroxy-2-methylpropyl)-1H-imidazo[4,5-c]quinolin-4-yl)amino)-6-oxohexyl)carbamate (30)

To an ice-chilled stirring solution of **6** (0.080 g, 0.254 mmol) in dry DMF (2 mL), **11** (0.118 g, 0.509 mmol), DIPEA (133 µL, 0.762 mmol), and HATU (0.194 g, 0.509 mmol) were added and stirred overnight at room temperature. The reaction mixture was diluted with ethyl acetate (20 mL) and washed with 1 M HCL (2 × 15 mL), saturated $NaHCO_3$ (2 × 15 mL), and brine. Organic phase was concentrated *in vacuo* to produce compound **30** as white solid. (115 mg, 86%). ^{1}H NMR (400 MHz, DMSO-d_6) δ 9.96 (s, 1H), 8.59 – 8.48 (m, 1H), 7.93 (dd, *J* = 8.3, 1.4 Hz, 1H), 7.68 – 7.57 (m, 1H), 7.58 – 7.46 (m, 1H), 6.87 – 6.67 (m, 1H), 4.93 (s, 3H), 4.84 – 4.62 (m, 2H), 3.62 – 3.48 (m, 1H), 3.05 – 2.77 (m, 3H), 2.77 – 2.58 (m, 2H), 1.69 – 1.54 (m, 1H), 1.53 – 1.28 (m, 13H), 1.23 – 1.07 (m, 9H).

2.3.24 Ethyl N^5-(6-((2-(ethoxymethyl)-1-(2-hydroxy-2-methylpropyl)-1H-imidazo[4,5-c]quinolin-4-yl)amino)-6-oxohexyl)-N^2-((E)-3-(4-hydroxy-3-methoxyphenyl)acryloyl)glycyl-L-valyl-D-glutaminate (32)

Compound **30** (0.115 g, 0.218 mmol) was deprotected using General procedure C. The resulting intermediate was dissolved in dry DMF (2 mL), chilled on ice and **5a** (0.142 g, 0.240 mmol), DIPEA (114 µL, 0.654 mmol), and HATU (0.092 g, 0.240 mmol) were added. The reaction mixture was stirred at room temperature overnight. Subsequently 1 M HCl (10 mL) was added and the reaction mixture was allowed to mix for 10 min, after which it was extracted with a mixture of DCM/isopropyl alcohol (3:1) (2 × 20 mL). The combined organic extracts were washed with saturated $NaCHO_3$ (2 × 15 mL) and brine (20 mL), dried over anhydrous Na_2SO_4 and concentrated *in vacuo*. The crude compound was purified using Isolera One flash chromatography (acetonitrile/0.1% TFA 20% → 100%) to obtain a compound **32** as a white solid (15 mg, 7%). ^{1}H NMR (400 MHz, DMSO-d_6) δ 9.96 (s, 1H), 9.45 (s, 1H), 8.53 (d, *J* = 8.4 Hz, 1H), 8.40 (d, *J* = 7.2 Hz, 1H), 8.20 (t, *J* = 5.7 Hz, 1H), 7.96 – 7.73 (m, 4H), 7.62 (t, *J* = 7.6 Hz, 1H), 7.54 (t, *J* = 7.6 Hz, 1H), 7.34 (d, *J* = 15.7 Hz, 1H), 7.15 (d, *J* = 2.0 Hz, 1H), 7.00 (dd, *J* = 8.1 Hz, 1H), 6.79

(d, *J* = 8.1 Hz, 1H), 6.57 (d, *J* = 15.7 Hz, 1H), 4.93 (s, 2H), 4.75 (s, 2H), 4.29 – 4.23 (m, 1H), 4.21 – 4.14 (m, 1H), 4.10 – 4.04 (m, 2H), 3.90 (d, *J* = 5.8 Hz, 2H), 3.82 – 3.69 (m, 4H), 3.59 – 3.48 (m, 2H), 3.07 – 2.99 (m, 3H), 2.17 – 2.07 (m, 3H), 2.04 – 1.91 (m, 4H), 1.87 – 1.76 (m, 2H), 1.68 – 1.60 (m, 2H), 1.47 – 1.33 (m, 6H), 1.20 – 1.10 (m, 12H), 0.88 – 0.79 (m, 9H). ^{13}C NMR (100 MHz, DMSO) δ 207.00, 172.57, 172.26, 171.49, 171.35, 169.50, 166.31, 152.59, 148.83, 148.28, 144.96, 143.74, 139.96, 136.17, 129.28, 129.22, 127.55, 126.79, 124.92, 122.28, 122.08, 118.96, 117.60, 116.11, 111.35, 71.14, 65.96, 65.36, 60.92, 57.87, 55.98, 55.45, 52.23, 42.75, 40.64, 40.59, 40.38, 40.17, 39.96, 39.75, 39.54, 39.33, 38.96, 36.55, 31.89, 31.24, 31.15, 29.48, 27.18, 26.68, 25.09, 19.65, 18.20, 15.46, 14.49. HRMS *m/z* calculated for $C_{47}H_{65}O_{11}N_8$: 917.4767 (M + H)$^+$, found 917.4753.

2.3.25 Benzyl (6-((1-(4-amino-2-(ethoxymethyl)-1H-imidazo[4,5-c]quinolin-1-yl)-2-methylpropan-2-yl)amino)-6-oxohexyl)carbamate (31)

To a solution of **7** (0.100 g, 0.319 mmol) in DMF (1.5 mL) were added **12** (0.089 g, 0.335 mmol) and DIPEA (122 µL, 0.702 mmol). The stirring solution was chilled on ice and HOBt (0.045 g, 0.335 mmol), a catalytic amount of DMAP, and EDC × HCl (0.064 g, 0.335 mmol) were added. The reaction mixture was stirred overnight at room temperature. Subsequently water (20 mL) was added and extracted with ethyl acetate (3 × 15 mL). Combined organic phases were washed with water (2 × 15 mL), brine (15 mL), and saturated $NaHCO_3$ (2 × 20 mL), dried over Na_sSO_4 and concentrated *in vacuo* to produce compound **31** as an orange oil (177 mg, 99%). ^{1}H NMR (400 MHz, DMSO-d_6) δ 8.34 (dd, *J* = 8.5, 1.4 Hz, 1H), 7.71 – 7.58 (m, 2H), 7.46 – 7.20 (m, 8H), 6.62 (s, 1H), 5.00 (s, 4H), 4.74 (s, 2H), 3.51 (q, *J* = 6.9, 6.2 Hz, 2H), 2.99 (q, *J* = 6.6 Hz, 2H), 2.12 – 2.00 (m, 2H), 1.53 – 1.34 (m, 5H), 1.29 – 1.05 (m, 10H).

2.3.26 Ethyl N^5-(6-((1-(4-amino-2-(ethoxymethyl)-1H-imidazo[4,5-c]quinolin-1-yl)-2-methylpropan-2-yl)amino)-6-oxohexyl)-N^2-((E)-3-(4-hydroxy-3-methoxyphenyl)acryloyl)glycyl-L-valyl-D-glutaminate (33)

A solution of **31** (0.165 g, 0.294 mmol) in ethanol (20 mL) was hydrogenated over 10% palladium-on-carbon overnight at room temperature and atmospheric pressure. The catalyst was removed by filtration and the filtrate concentrated *in vacuo*. The residual was washed with ether and dried. The resulting solid was dissolved in DMF (2.5 mL) and **5a** (0.122 g, 0.206 mmol) DIPEA (90 µL, 0.516 mmol) and COMU (0.099 g, 0.232 mmol) were added on ice. The reaction mixture was stirred overnight at room temperature. Subsequently 1 M HCl (10 mL) were added and the reaction mixture was stirred for 15 minutes, after which the product was extracted with DCM (2 × 30 mL). Combined organic phases were washed with saturated $NaHCO_3$ (2 × 30 mL), brine (30 mL), dried over Na_sSO_4, and concentrated *in vacuo*. Resulting mixture was purified using Isolera One flash chromatography (acetonitrile/0.1% TFA 20% → 100%) to obtain compound **33** as a yellow solid (15 mg, 8%). ^{1}H NMR (400 MHz, DMSO-d_6) δ 9.43 (s, 1H), 8.44 – 8.27 (m, 2H), 8.19 (t, *J* = 5.7 Hz, 1H), 7.86 (d, *J* = 9.0 Hz, 1H), 7.81 – 7.71 (m, 1H), 7.71 – 7.54 (m, 2H), 7.47 – 7.38 (m, 1H), 7.34 (d, *J* = 15.7 Hz, 1H), 7.30 – 7.17 (m, 1H), 7.15 (d, *J* = 2.0 Hz, 1H), 7.06 – 6.98 (m, 1H), 6.79 (d, *J* = 8.1 Hz, 1H), 6.56 (d, *J* = 15.7 Hz, 2H), 5.00 (s, 2H), 4.74 (s, 2H), 4.30 – 3.98 (m, 4H), 3.90 (d, *J* = 5.7 Hz, 2H), 3.80 (s, 3H), 3.59 – 3.46 (m, 2H), 3.07 – 2.96 (m, 2H), 2.17 – 2.09 (m, 2H), 2.06 – 1.93 (m, 4H), 1.83 – 1.73 (m, 1H), 1.52 – 1.32 (m, 5H), 1.27 – 1.04 (m, 18H), 0.84 (t, *J* = 6.9 Hz, 6H). HRMS *m/z* calculated for $C_{47}H_{66}O_{10}N_9$: 916.4927 (M + H)$^+$, found 916.4907.

2.4 Solubility measurement

The kinetic solubilities of conjugates were estimated using the method described by Hoelke et al.[3] Briefly, in duplicate, a 10 mM solution of compound in DMSO was diluted with pH 7.4 phosphate-buffered saline (PBS) to give a final DMSO concentration of 5%. After 15 minutes, the resulting

suspension was filtered through a syringe filter (0.45 μm), diluted 2.25-fold with acetonitrile, and analyzed with UHPLC. The solubility was quantified with a five-point calibration curve from 500 to 0.8 µM generated by dilution of the original DMSO solution into a 1:1 mixture of acetonitrile and PBS. The content of DMSO in all solutions was fixed to 5% by adding the respective amounts of DMSO. The column used was Waters Acquity UPLC BEH C18 (1.7 µm, 2.1 × 50 mm). The eluent was a mixture of 0.1% TFA in water (A) and acetonitrile (B), with a gradient of (%B): 0−6 min, 5−95%; 6−8 min, 95%; 8−8.5 min, 95−5%. The column was thermostated at 40 °C.

3. Biology

3.1. Hek-Blue NOD2, TLR7 and TLR8 cells.

HEK-Blue NOD2, TLR7, and TLR8 cells (Invivogen, San Diego, CA, USA) were cultured according to the manufacturer instructions in Dulbecco's modified Eagle's medium (Sigma-Aldrich, St. Louis, MO, USA) supplemented with 10% heat-inactivated fetal bovine serum (Gibco), 2 mM L-glutamine (Sigma-Aldrich), 100 U/mL penicillin (Sigma-Aldrich), 100 µg/mL streptomycin (Sigma-Aldrich), and 100 µg/mL Normocin (Invivogen) for two passages. All subsequent passages of NOD2 and TLR8 cells were cultured in medium additionally supplemented with 100 µg/mL Zeocin and 30 µg/mL Blasticidin (Invivogen) and of TLR7 cells with 100 µg/mL Zeocin and 10 µg/mL Blasticidin. The cells were incubated in a humidified atmosphere at 37 °C and 5 % CO_2.

3.2 Peripheral Blood Mononuclear Cells

Human PBMCs from healthy and consenting donors were isolated from heparinized blood by density gradient centrifugation with Ficoll-Paque (Pharmacia, Sweden). The isolated cells were washed twice with PBS, resuspended in RPMI 1640 medium (Sigma-Aldrich, St. Louis, MO, USA) supplemented with 10% heat-inactivated fetal bovine serum (Gibco), 2 mM L-glutamine (Sigma-Aldrich), 100 U/mL penicillin (Sigma-Aldrich), and 100 µg/mL streptomycin (Sigma-Aldrich), and used in the assays.

3.3 Cytotoxicity

The tested compounds were dissolved in DMSO and further diluted in culture medium to the desired final concentrations, such that the final DMSO concentration never exceeded 0.1%. HEK-Blue NOD2, TLR7, and TLR8 cells were seeded (40,000 cells/well) in 96-well plates in 100 µL of culture medium, and treated with 10 µM of each compound or with the corresponding vehicle (0.1% DMSO; control cells). After 18 h of incubation (37 °C, 5 % CO_2), the metabolic activity was assessed using the CellTiter 96 Aqueous One Solution cell proliferation assay (Promega, Madison, WI, USA), according to the manufacturer instructions. The experiments were run in duplicates, and repeated as two independent biological replicates. Statistical significance was determined with one-way ANOVA with subsequent Dunnett's multiple comparisons test.

3.4 NOD2/TLR7/TLR8 NF-kB reporter assay

HEK-Blue NOD2 cells (25,000 cells/well), TLR7 and TLR8 cells (40,000 cells/well) were seeded in 96-well plates in 100 µL HEK-Blue detection medium (Invivogen, San Diego, CA, USA) and treated with compounds (8 different concentrations from 0.6 nM to 10 µM (for EC_{50} determination) or with the corresponding vehicle (0.1% DMSO). After 18 h of incubation (37 °C, 5% CO_2), secreted embryonic alkaline phosphatase (SEAP) activity was determined spectrophotometrically as absorbance at 630 nm (BioTek Synergy microplate reader; Winooski, VT, USA). EC_{50} values were calculated using Prism

software (version 9; GraphPad Software, CA, USA). The experiments were run in duplicates, and repeated as at least three independent biological replicates.

3.5 Cytokine Release from Peripheral Blood Mononuclear Cells

Peripheral blood mononuclear cells were seeded (1 × 10^6 cells/mL) in 96-well plates in 100 µL of growth medium and treated with the compounds (1 µM), or the corresponding vehicle (0.1% DMSO). Cell-free supernatants were collected after 18 h of incubation (37 °C, 5% CO_2) and stored at −80 °C until tested. Cytokine concentrations were determined with the LEGENDplex™ HU Essential Immune Response Panel (Biolegend) on an Attune NxT flow cytometer (Thermo Fisher Scientific, Waltham, MA). Standard curves were generated using recombinant cytokines contained in the kit. The data were analyzed using the FlowJo (Tree Star, Inc., Ashland, OR) and Prism (GraphPad, San Diego, CA) software. Statistical significance was determined with one-way ANOVA with subsequent Dunnett's multiple comparisons test.

3.6 Statistics

The data were analyzed using Prism software (version 9; GraphPad Software, CA, USA). Statistical significance was determined according to the specific procedures outlined in each experiment. A p-value < 0.05 was considered statistically significant.

4. Representative NMR Spectra

Compound **22**: ^{1}H, 400 MHz, DMSO-d_6

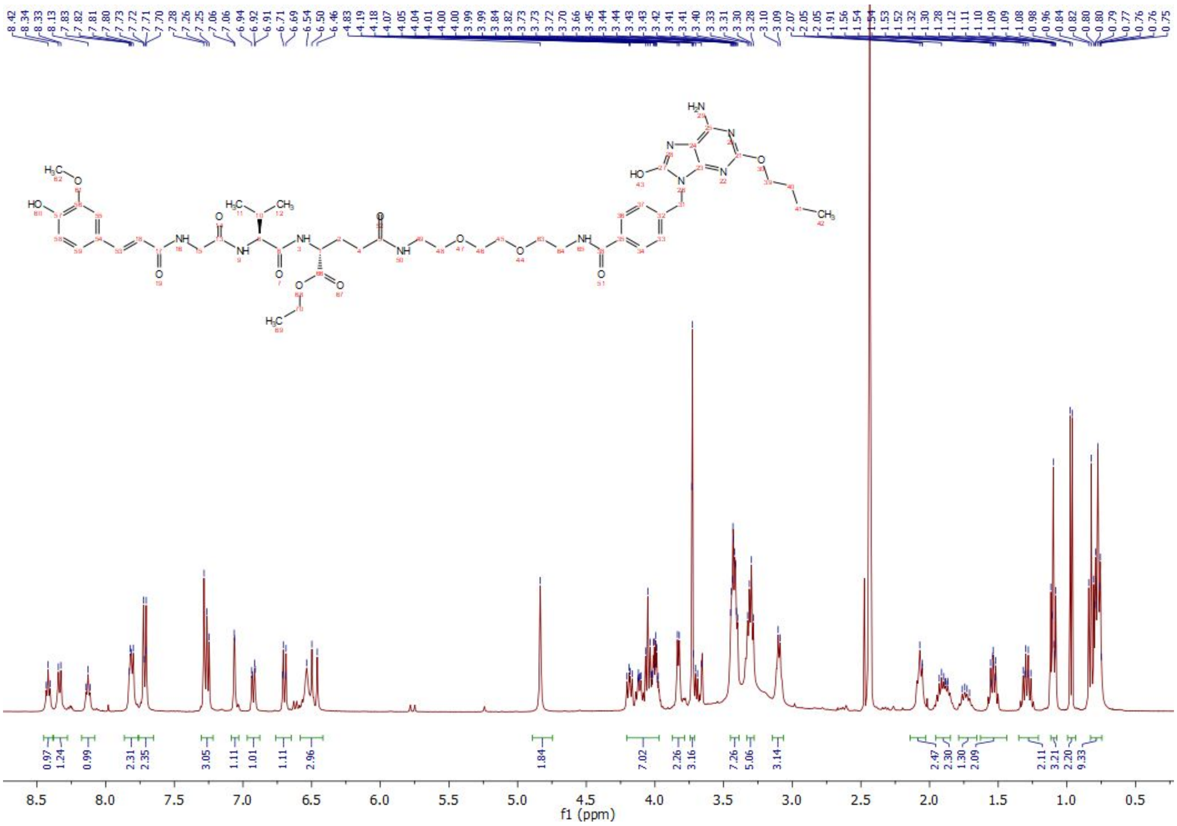

Compound **22**: ^{13}C, 100 MHz, DMSO-d_6

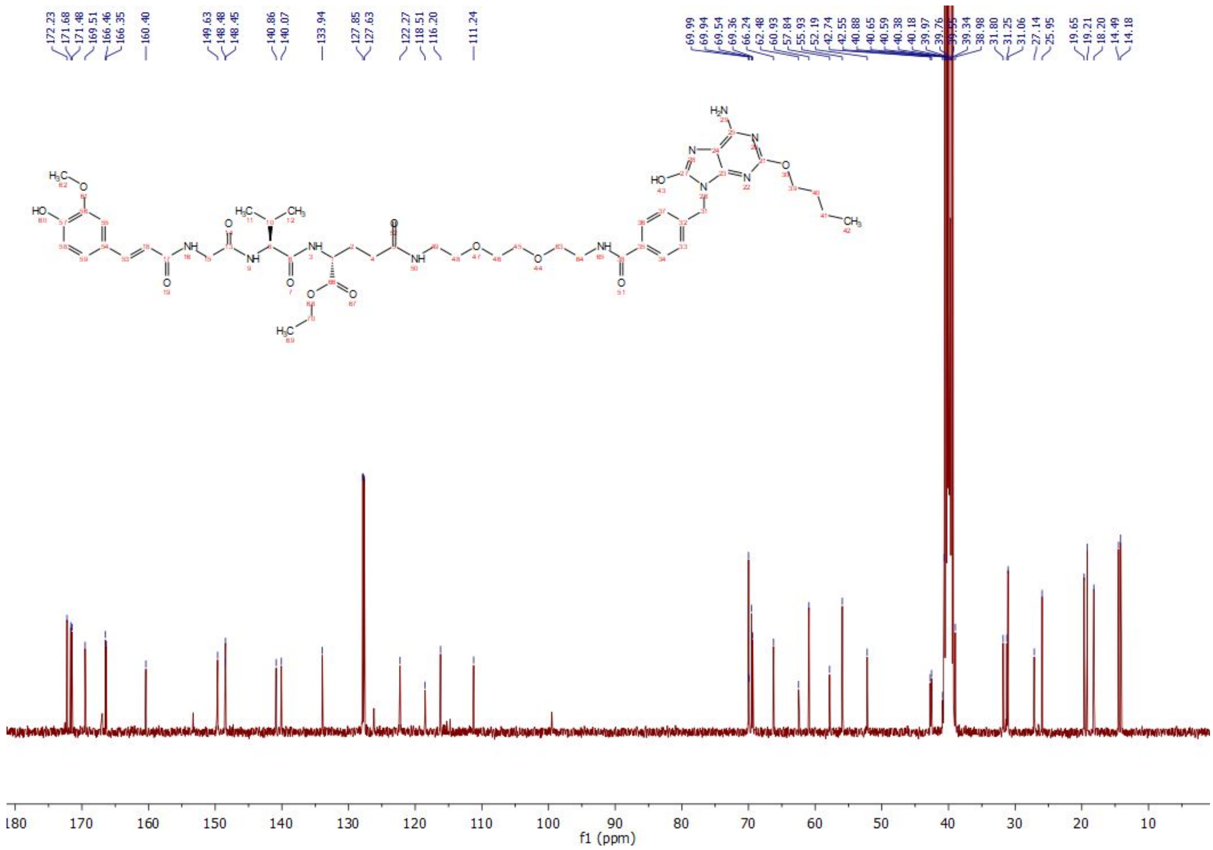

Compound **23**: ^{1}H, 400 MHz, DMSO-d_6

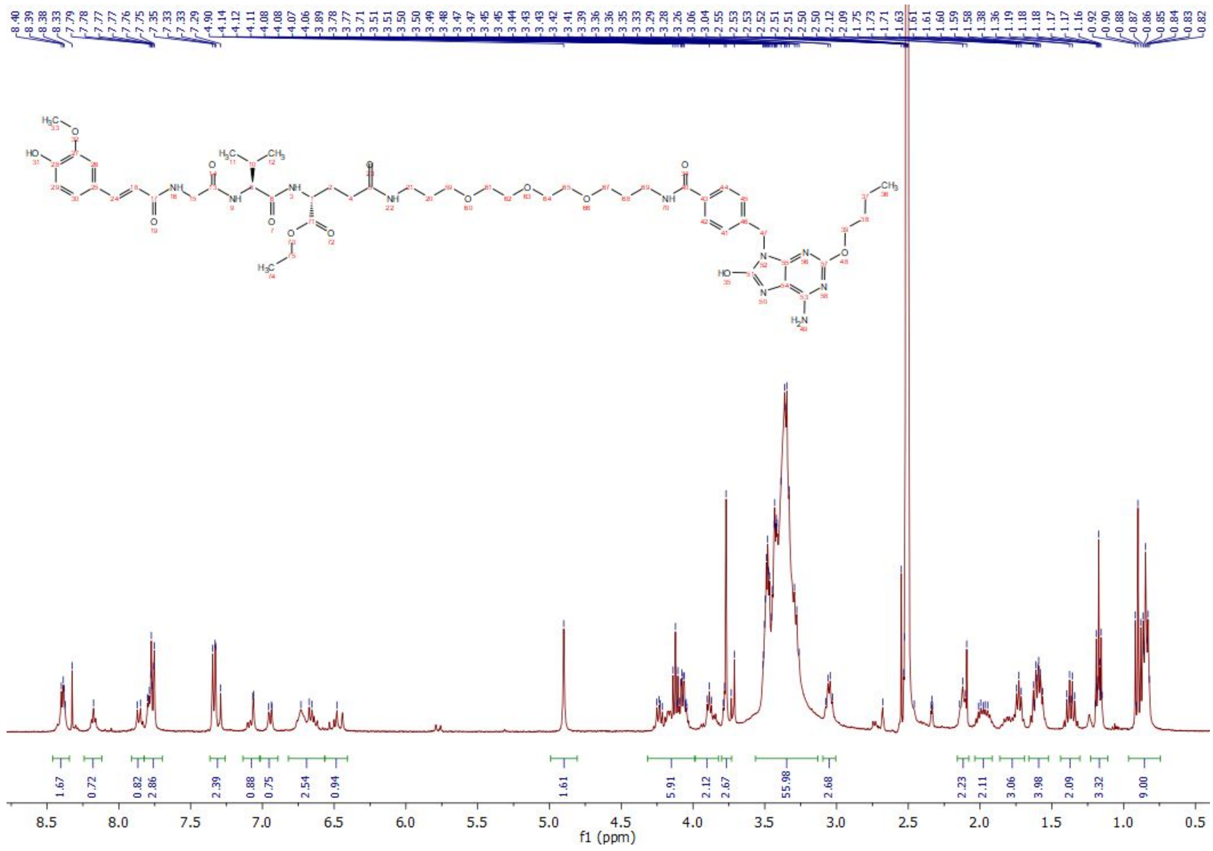

Compound **24**: ^{1}H, 400 MHz, DMSO-d_6

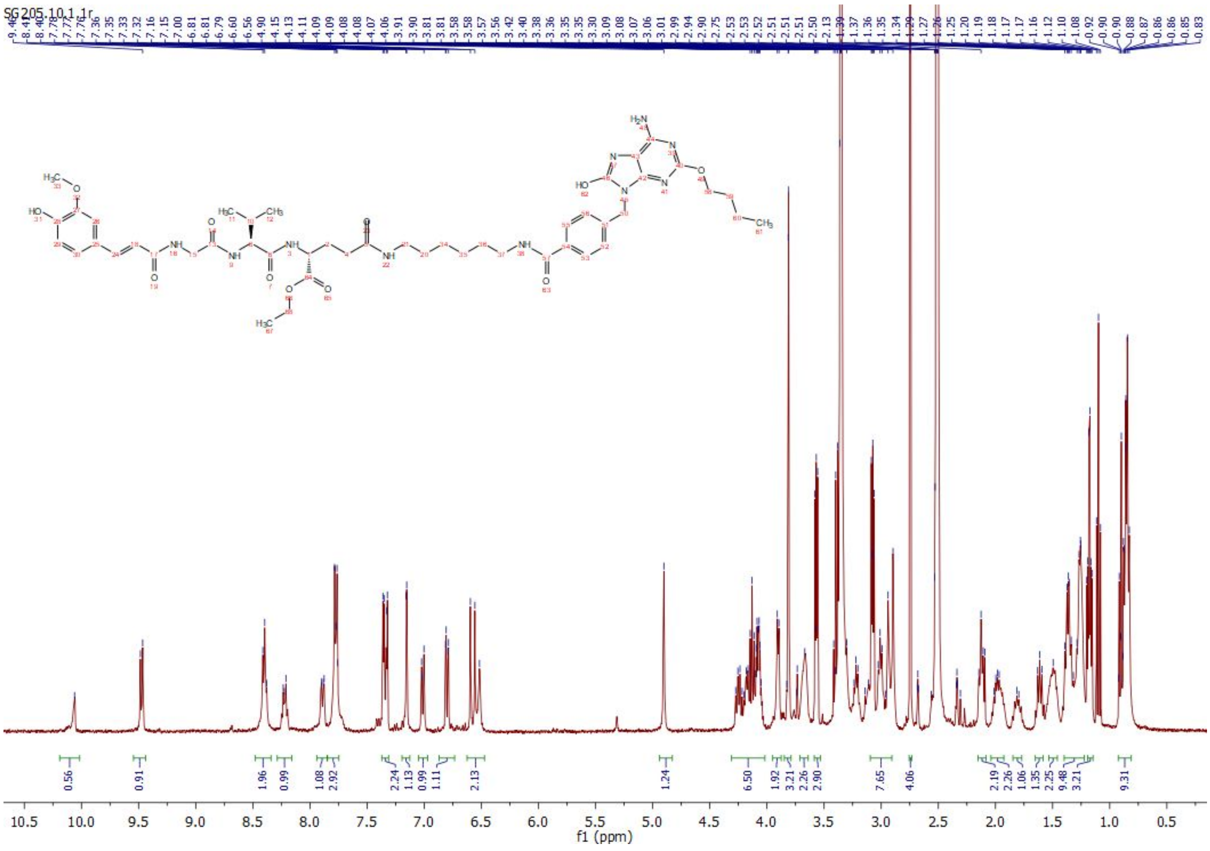

Compound **26**: ^{1}H, 400 MHz, DMSO-d$_6$

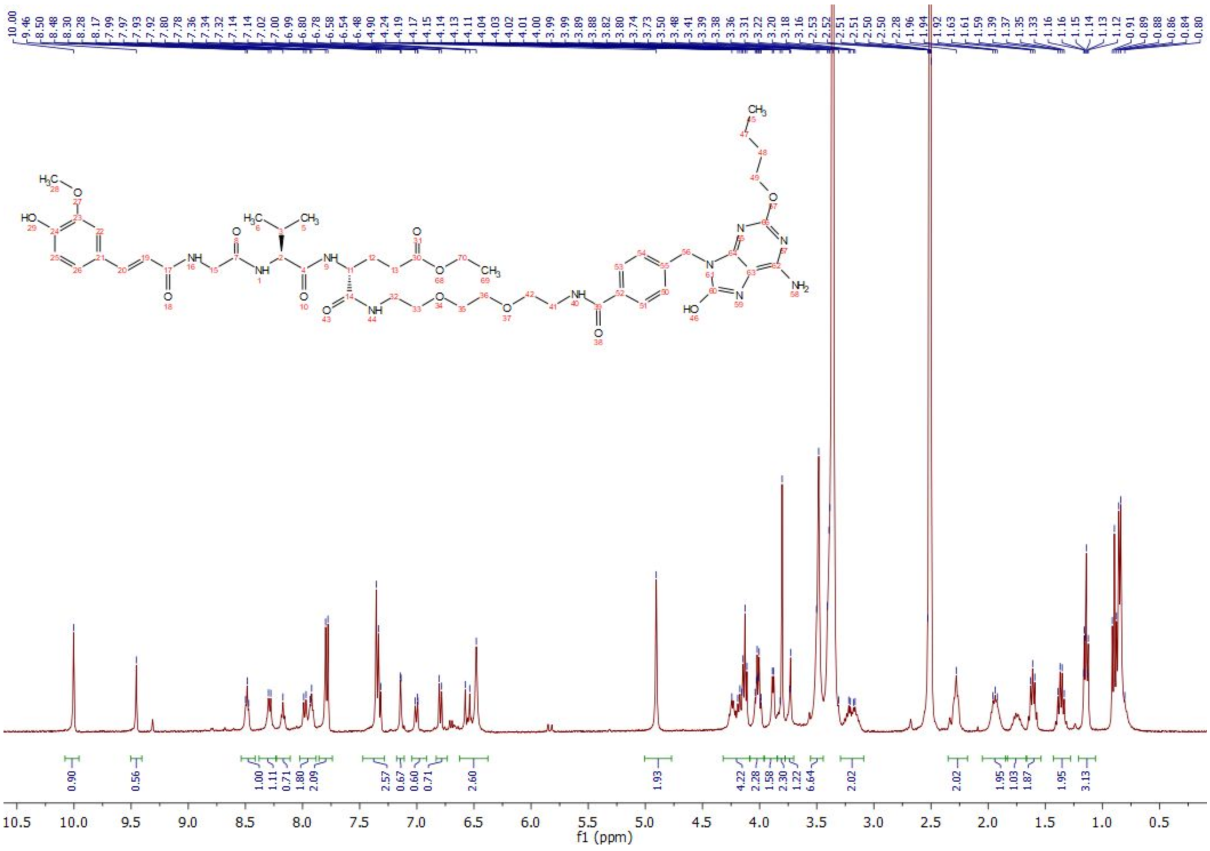

Compound **29**: ^{1}H, 400 MHz, DMSO-d$_6$

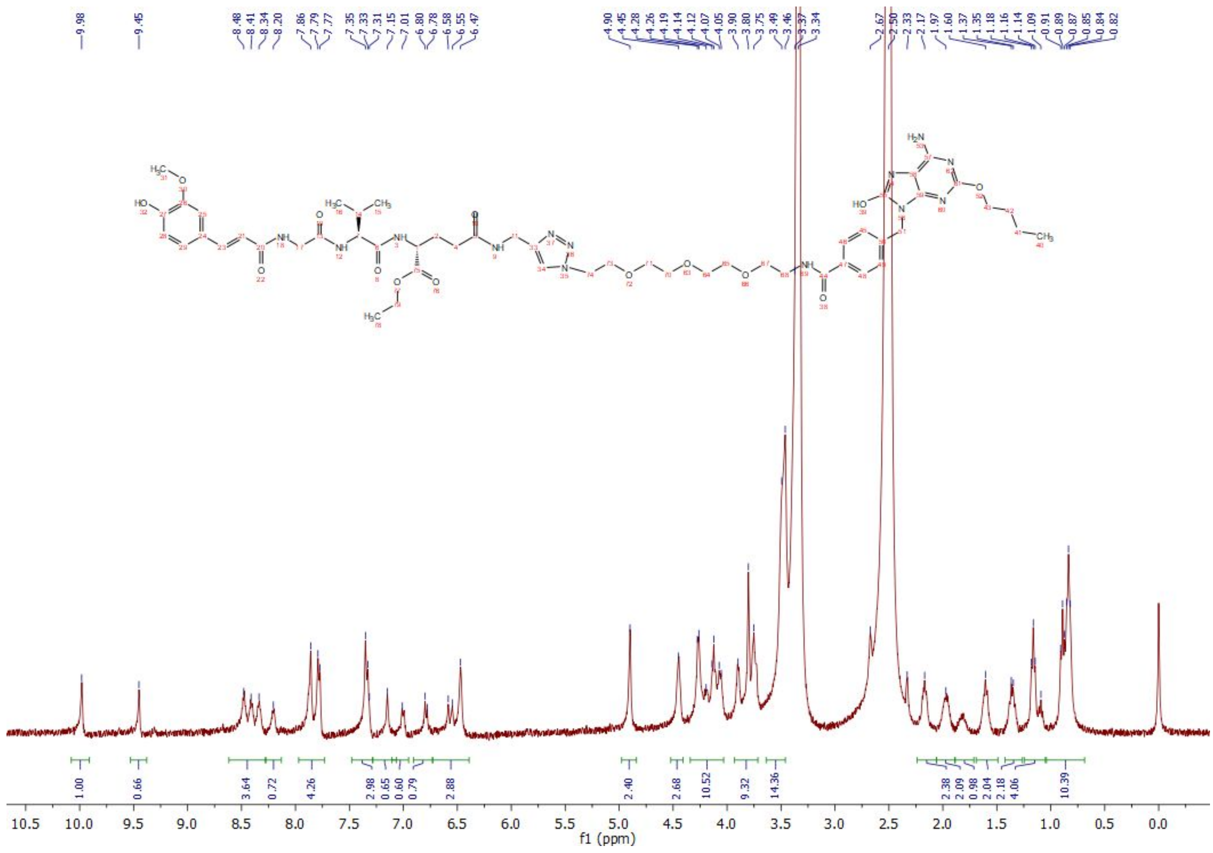

Compound **32**: ^{1}H, 400 MHz, DMSO-d$_6$

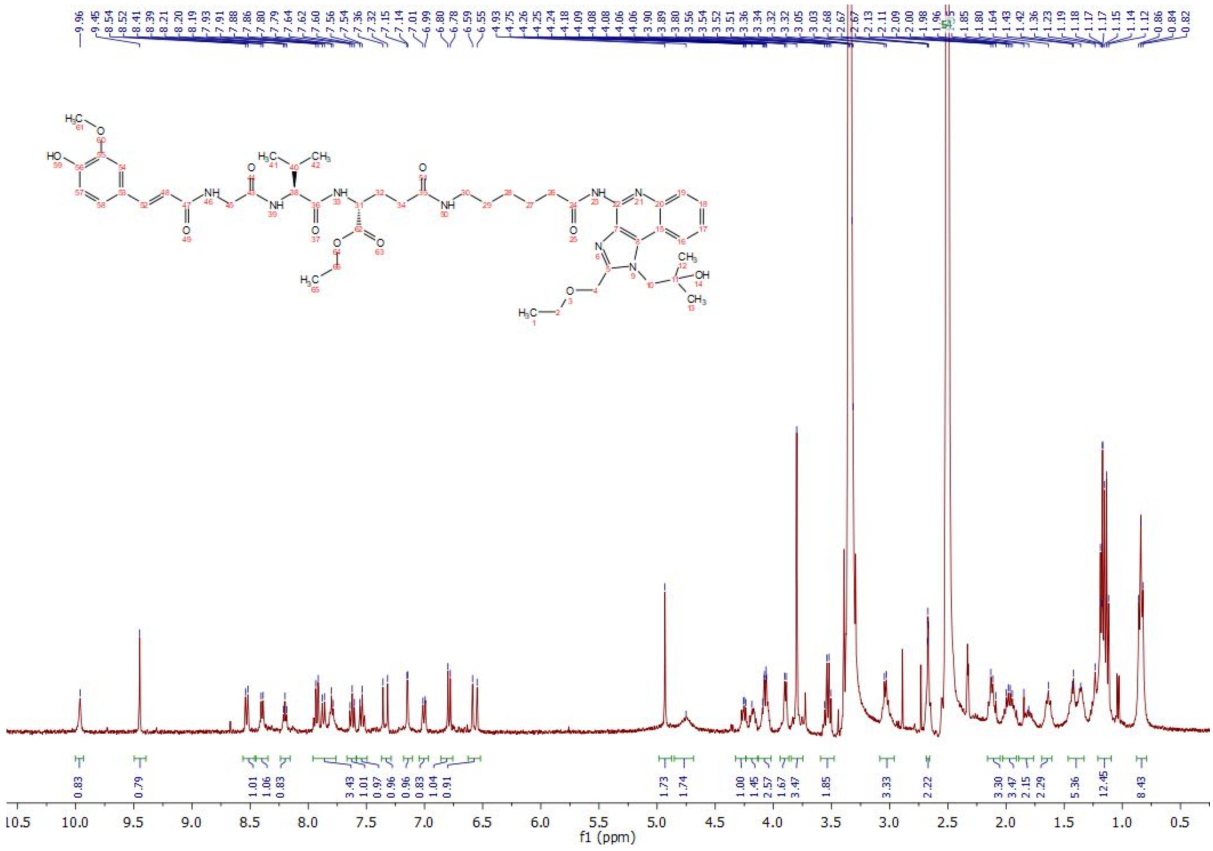

Compound **32**: ^{13}C, 100 MHz, DMSO-d$_6$

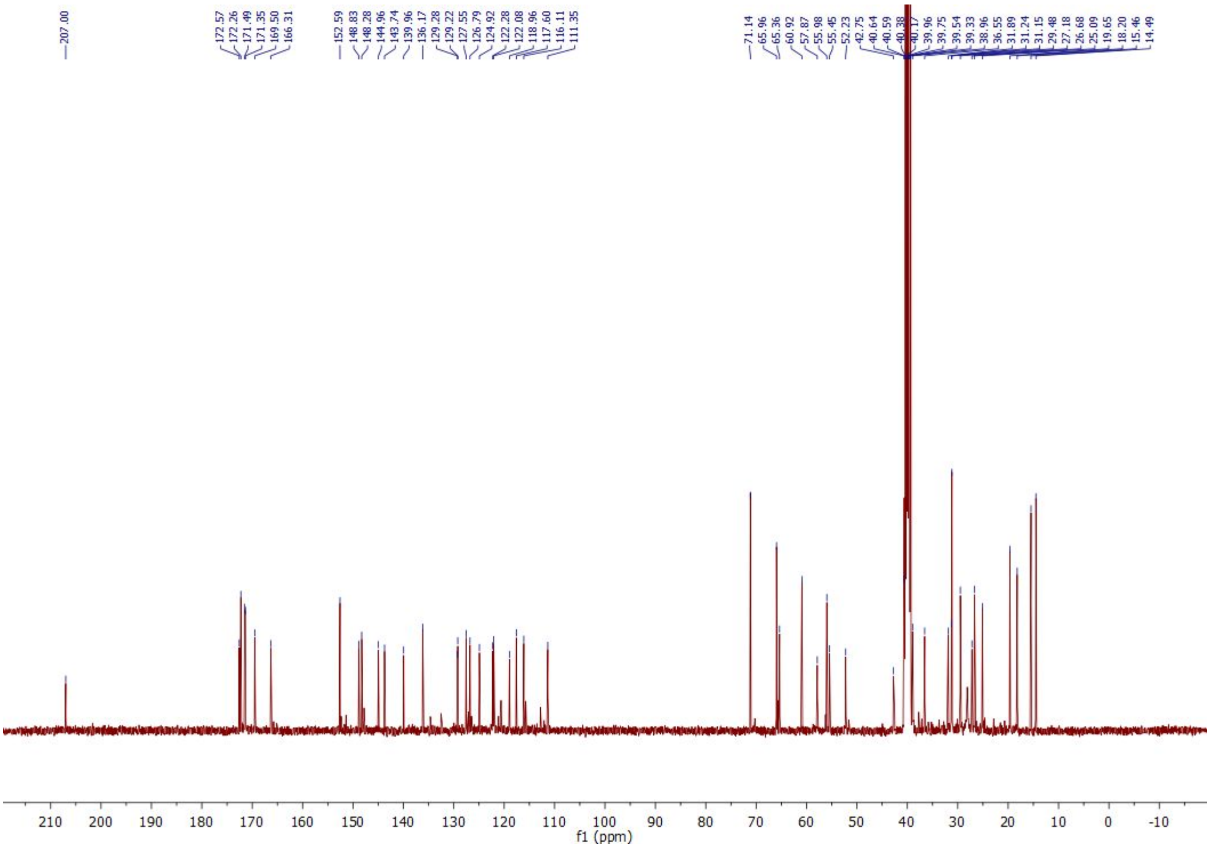

Compound **33**: ^{1}H, 400 MHz, DMSO-d_6

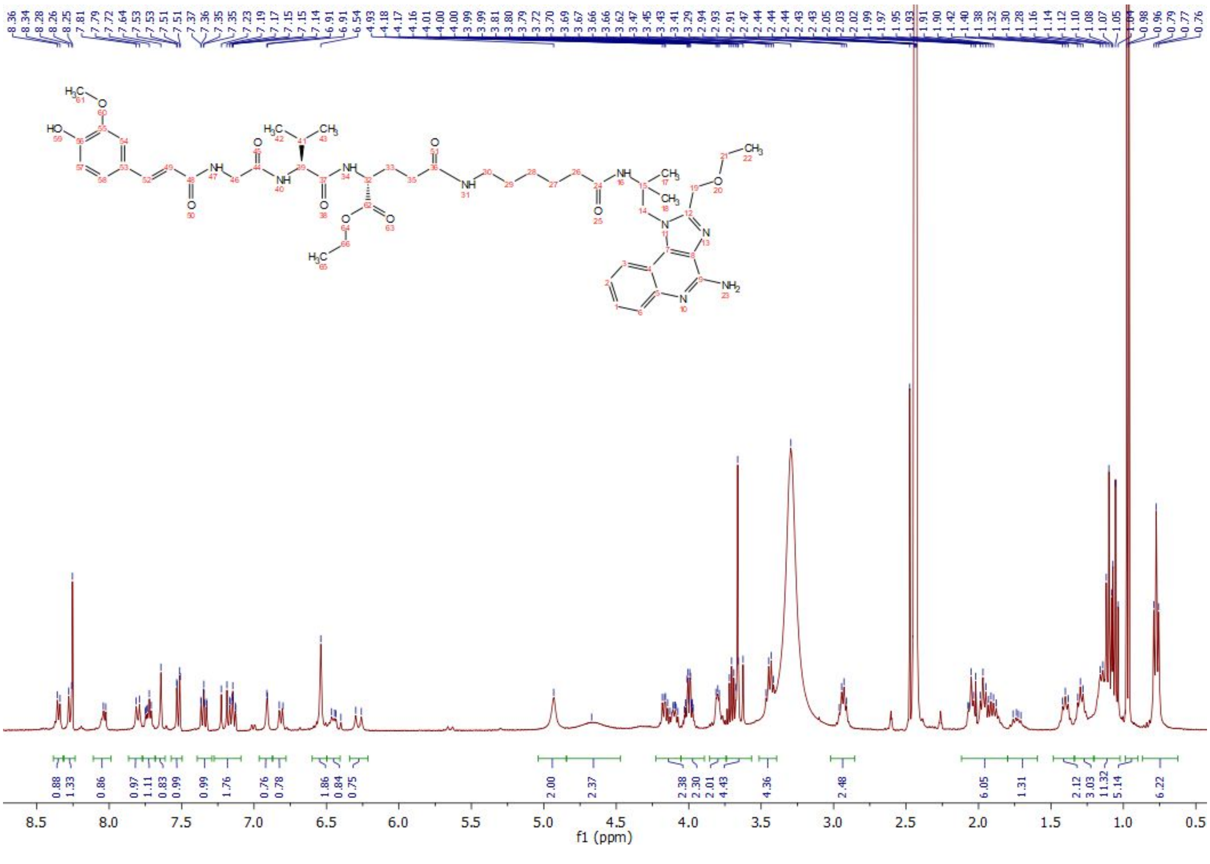

5. Representative UHPLC traces

Compound **23**

Chromatogram and Results

Injection Details

Processing Method:	**Quantitative**	*Run Time (min):*	**12,50**
		Wavelength:	**254**

Chromatogram

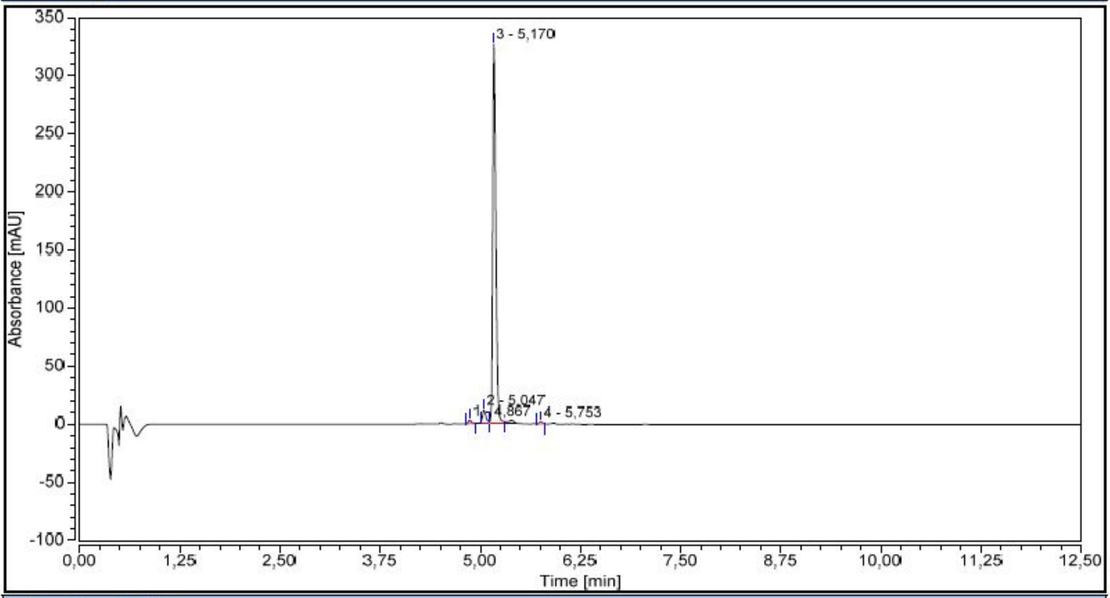

Integration Results

No.	Peak Name	Retention Time min	Area mAU*min	Height mAU	Relative Area %	Relative Height %	Amount n.a.
1		4,867	0,134	3,102	0,89	0,91	n.a.
2		5,047	0,533	10,964	3,52	3,20	n.a.
3		5,170	14,388	326,275	95,08	95,34	n.a.
4		5,753	0,077	1,883	0,51	0,55	n.a.
Total:			**15,133**	**342,224**	**100,00**	**100,00**	

Compound **24**

Chromatogram and Results

Injection Details

Processing Method:	**Quantitative**	*Run Time (min):*	**12,50**
		Wavelength:	**254**

Chromatogram

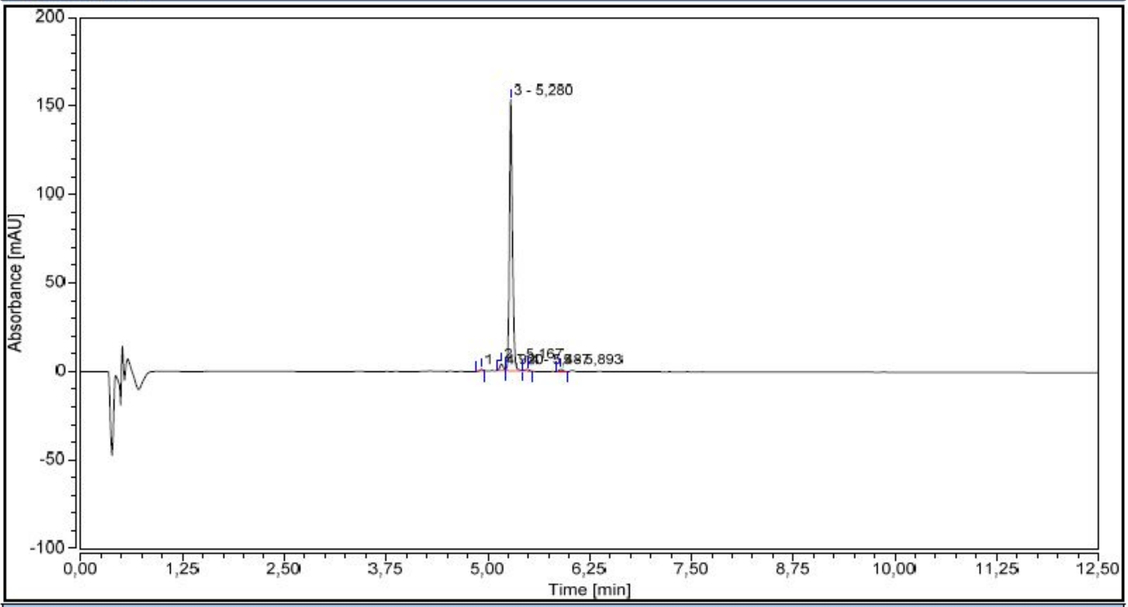

Integration Results

No.	Peak Name	Retention Time min	Area mAU*min	Height mAU	Relative Area %	Relative Height %	Amount n.a.
1		4,920	0,034	0,935	0,49	0,58	n.a.
2		5,167	0,183	4,045	2,63	2,52	n.a.
3		5,280	6,616	153,122	95,03	95,45	n.a.
4		5,487	0,066	0,971	0,95	0,61	n.a.
5		5,893	0,063	1,341	0,90	0,84	n.a.
**Total:**			**6,962**	**160,414**	**100,00**	**100,00**	

Compound **32**

Chromatogram and Results

Injection Details

Processing Method:	Quantitative	*Run Time (min):*	12,50
		Wavelength:	254

Chromatogram

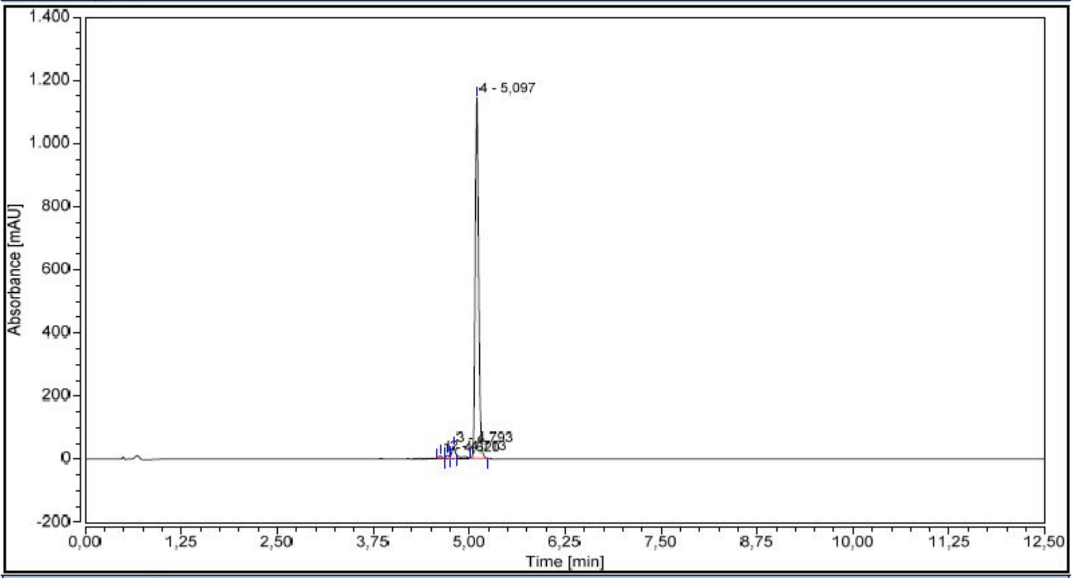

Integration Results

No.	Peak Name	Retention Time min	Area mAU*min	Height mAU	Relative Area %	Relative Height %	Amount n.a.
1		4,620	0,415	9,218	0,68	0,77	n.a.
2		4,713	0,563	11,383	0,92	0,95	n.a.
3		4,793	1,948	38,195	3,18	3,18	n.a.
4		5,097	58,234	1141,650	95,22	95,10	n.a.
Total:			**61,159**	**1200,445**	**100,00**	**100,00**	

6. Supporting table

	IL-1β	IL-2	IL-4	IL-6	IL-8	IL-10	IL-12p70	IL-17A	TNF-α	IFN-γ	MCP-1	IP-10	TGF-β1
4	8.05 ± 4	0.77 ± 0.6	1.11 ± 0.4	171.47 ± 78.6	496.54 ± 188.2	5.45 ± 2.6	1.2 ± 0.2	1.84 ± 0.6	14.16 ± 8.2	3.16 ± 0.8	80.87 ± 36.9	94.07 ± 10.2	3.86 ± 1.9
5	65.6 ± 11.7	1 ± 0.5	3.2 ± 1.1	1120.03 ± 204.2	10359.47 ± 4469.2	4.5 ± 2.4	2.13 ± 1	2 ± 1	96.8 ± 17.6	21.97 ± 18.5	1118.43 ± 124.2	116.83 ± 4.8	7.9 ± 3.8
4+5	189.47 ± 20.6	0.47 ± 0.4	7.23 ± 1.2	2631.7 ± 101.4	9267.3 ± 3671.6	7.73 ± 1.9	4.83 ± 2	2.2 ± 0.6	303.57 ± 117.1	161.23 ± 154.5	2717.23 ± 998.3	110.9 ± 7.9	12.7 ± 1.9
1	481.27 ± 145.4	2.5 ± 1.4	21.6 ± 0.6	6589.53 ± 2094.6	3833.73 ± 796.7	29.47 ± 1.4	27.23 ± 8.9	3.13 ± 0.3	596.3 ± 273.4	1228.7 ± 408.4	7611.03 ± 96.9	562.67 ± 232.7	35.57 ± 0.6
2	1477.4 ± 36.2	1.5 ± 0.1	25.37 ± 0.9	12848.07 ± 363.9	17564.5 ± 2576.7	88.2 ± 18.7	133.5 ± 36.6	3.93 ± 0.3	3838.37 ± 135.9	2666.5 ± 158.1	8497.8 ± 225.3	1391.43 ± 158	82.27 ± 1.4
22	142.95 ± 32.6	7.27 ± 2.9	15.53 ± 0.9	4768.76 ± 359.6	1763.4 ± 523.5	131.04 ± 87.5	30.12 ± 8.7	3.71 ± 0.6	564.4 ± 216.4	133.16 ± 28.2	8735.94 ± 718.5	1461.02 ± 119.4	23.54 ± 3.7
23	620.46 ± 150.3	2.85 ± 2.1	19.52 ± 1.5	8121.83 ± 468.1	3223.31 ± 946.2	167.01 ± 106.5	68.86 ± 26.8	3.52 ± 0.8	1396.53 ± 518.2	1179.93 ± 325.7	9010.74 ± 723.7	1226.09 ± 285	44.85 ± 7.2
24	896.08 ± 177.4	2.31 ± 0.9	21.97 ± 0.3	10885.9 ± 781.2	7067.18 ± 2792.6	184.61 ± 111.4	113.6 ± 46.8	3.67 ± 0.7	2122.87 ± 712.8	2257.6 ± 613.2	9674.94 ± 1662.3	1357.54 ± 237.9	61.64 ± 8.9
26	57.5 ± 14.3	3.17 ± 1.5	15.93 ± 0.7	3972.97 ± 505.8	1184.9 ± 264.6	66.77 ± 26.3	14.27 ± 3.4	2.47 ± 0.4	344.9 ± 89.9	49.9 ± 11.7	9380.27 ± 992.8	1801.67 ± 614.9	12.3 ± 1.3
29	38.86 ± 17.5	4.13 ± 1.6	8.58 ± 4.8	3450.43 ± 1457.9	152.03 ± 90.4	51.5 ± 10.7	18.91 ± 10.3	1.18 ± 0.8	162.82 ± 80.2	161.77 ± 101.4	5007.01 ± 1552.5	762.74 ± 202.7	6.03 ± 6
6	695.88 ± 41.1	1.36 ± 0	1.71 ± 0.2	15405.79 ± 1354	1482.12 ± 247.5	54.59 ± 0.8	25.47 ± 1.4	0.29 ± 0.1	891.44 ± 66.3	649.17 ± 40.6	5505.9 ± 31.5	839.46 ± 89.7	0 ± 0
7	267.72 ± 20.1	1.51 ± 0.3	1.46 ± 0.1	4799.41 ± 150.5	411.48 ± 49.4	28.73 ± 0.8	16.23 ± 1.6	0.27 ± 0	402.66 ± 12.8	287.14 ± 3.4	5896.77 ± 56.4	718.32 ± 229.2	0 ± 0
6+5	1887.16 ± 81.5	1.43 ± 0.1	1.79 ± 0	41899.95 ± 1199.4	3950.84 ± 342.9	42.22 ± 2.8	45.82 ± 2.3	0.56 ± 0.2	3176.92 ± 143.6	1693.46 ± 111.4	5653 ± 81.7	1086.18 ± 26	2.38 ± 0.1
7+5	1206.1 ± 55.2	1.38 ± 0	1.79 ± 0.1	27286.79 ± 780.5	3476.7 ± 672.7	23.81 ± 2.2	37.07 ± 2.4	0.34 ± 0	1965.25 ± 92.2	932.27 ± 50.7	5563.92 ± 292.9	670.9 ± 68.1	0.92 ± 0.2
32	0 ± 0	0.17 ± 0	0.38 ± 0.1	1.83 ± 0.5	9.63 ± 1	0.37 ± 0.1	0 ± 0	0 ± 0	0.25 ± 0	0 ± 0	9.59 ± 1.9	2.25 ± 0.3	0 ± 0
33	0 ± 0	0.4 ± 0.1	0.33 ± 0	33.23 ± 6.9	38.53 ± 5	1.4 ± 0.1	0 ± 0	0 ± 0	2.67 ± 1	0.12 ± 0.1	987.36 ± 453.3	379.87 ± 193	0 ± 0

Data are mean ± SEM of three independent experiments.

7. Abbreviations

- COMU - (1-Cyano-2-ethoxy-2-oxoethylidenaminooxy)dimethylamino-morpholino-carbenium hexafluorophosphate
- DCM - dichloromethane
- DIPEA - *N*,*N*-diisopropylethylamine
- DMAP - 4-dimethylaminopyridine
- DMF - dimethylformamide
- EDC - 1-ethyl-3-(3-(dimethylamino)propyl)carbodiimide
- HOBT - 1-hydroxybenzotriazole
- NOD - nucleotide-binding oligomerization domain
- PBMC - peripheral blood mononuclear cell
- TEA - triethylamine
- TFA - trifluoroacetic acid
- TLR - Toll-like receptor

8. References

1. Guzelj, S.; Weiss, M.; Slütter, B.; Frkanec, R.; Jakopin, Ž. Covalently Conjugated NOD2/TLR7 Agonists Are Potent and Versatile Immune Potentiators. *J. Med. Chem.* **2022**, *65* (22), 15085–15101.
2. Kedl, R. M. Immunostimulatory compositions and methods of stimulating an immune response. US 20040091491 A1, 2014.
3. Hoelke, B.; Gieringer, S.; Arlt, M.; Saal, C. Comparison of Nephelometric, UV-Spectroscopic, and HPLC Methods for High-Throughput Determination of Aqueous Drug Solubility in Microtiter Plates. *Anal. Chem.* **2009**, *81* (8), 3165–3172.
